# The HIF1α/HIF2α-miR210-3p network regulates glioblastoma cell proliferation, dedifferentiation and chemoresistance through EGF under hypoxic conditions

**DOI:** 10.1038/s41419-020-03150-0

**Published:** 2020-11-18

**Authors:** Pan Wang, Qian Yan, Bin Liao, Lu Zhao, Shuanglong Xiong, Junwei Wang, Dewei Zou, Jinyu Pan, Liangqi Wu, Yangmin Deng, Nan Wu, Sheng Gong

**Affiliations:** 1 Department of Neurosurgery, Chongqing General Hospital, University of Chinese Academy of Sciences, 401147 Chongqing, China; 2 Chongqing Medical University, 400016 Chongqing, China; 3 Department of Oncology, Chongqing University Cancer Hospital, 400030 Chongqing, China; 4 Chongqing Institute of Green and Intelligent Technology, Chinese Academy of Sciences, 400714 Chongqing, China; 5 University of Chinese Academy of Sciences, 100049 Beijing, China

**Keywords:** Cancer microenvironment, Cancer stem cells, CNS cancer

## Abstract

Hypoxia-inducible factor 1α (HIF1α) promotes the malignant progression of glioblastoma under hypoxic conditions, leading to a poor prognosis for patients with glioblastoma; however, none of the therapies targeting HIF1α in glioblastoma have successfully eradicated the tumour. Therefore, we focused on the reason and found that treatments targeting HIF1α and HIF2α simultaneously increased tumour volume, but the combination of HIF1α/HIF2α-targeted therapies with temozolomide (TMZ) reduced tumourigenesis and significantly improved chemosensitization. Moreover, miR-210-3p induced HIF1α expression but inhibited HIF2α expression, suggesting that miR-210-3p regulates HIF1α/HIF2α expression. Epidermal growth factor (EGF) has been shown to upregulate HIF1α expression under hypoxic conditions. However, in the present study, in addition to the signalling pathways mentioned above, the upstream proteins HIF1α and HIF2α have been shown to induce EGF expression by binding to the sequences AGGCGTGG and GGGCGTGG. Briefly, in a hypoxic microenvironment the HIF1α/HIF2α-miR210-3p network promotes the malignant progression of glioblastoma through a positive feedback loop with EGF. Additionally, differentiated glioblastoma cells underwent dedifferentiation to produce glioma stem cells under hypoxic conditions, and simultaneous knockout of HIF1α and HIF2α inhibited cell cycle arrest but promoted proliferation with decreased stemness, promoting glioblastoma cell chemosensitization. In summary, both HIF1α and HIF2α regulate glioblastoma cell proliferation, dedifferentiation and chemoresistance through a specific pathway, which is important for glioblastoma treatments.

## Introduction

Glioblastoma (GBM) undergoes malignant progression under hypoxic conditions^1,2^, which are mainly regulated by HIF1α and HIF2α^3–6^. Both HIF1α and HIF2α initially regulate the malignant progression of GBM^7^, but as the tumour progresses, the effect of HIF2α on tumour growth decreases, while HIF1α becomes increasingly important^8^. These results lead to the development of drugs targeting of HIF1α, aiming to inhibit GBM growth in patients^9–11^. Unfortunately, this targeted therapy has not been successful, since it is unable to remarkably reduce the tumour volume. Therefore, we individually or simultaneously knocked out HIF1α and HIF2α to determine the reason.

Importantly, hypoxia-related miRNAs have key functions in the malignancy of tumour^12,13^, which exhibit altered expression under hypoxic conditions, thus regulating the malignant progression of GBM^14,15^. By examining the hypoxia-related miRNAs in glioma, researchers have shown that miR-210-3p may be related to tumour growth through a HIF1α-dependent mechanism^16,17^. However, as shown in our study, a mutual regulatory feedback loop exists between HIF1α/HIF2α and miR210-3p and subsequently contributes to GBM malignant progression.

Epidermal growth factor (EGF) is expressed at high levels under hypoxic conditions^18^ and regulates GBM growth through EGFR and PI3K/AKT signalling pathways^19,20^. A meaningful observation is that one of the downstream genes of PI3K/AKT signalling pathway is HIF1α^21^, and many studies have confirmed that HIF1α expression depends on EGF^18,19^. Therefore, the mechanism of GBM growth under hypoxic conditions defined by previous studies is that EGF present at high levels binds to EGFR, which then activates the PI3K/AKT signalling pathway and induces steady expression of HIF1α to promote the malignant progression of the tumour^19^. However, few studies have directly assessed the mutual relationship between HIF1α/HIF2α and EGF, and we identified both HIF1α and HIF2α as upstream factors that contribute to regulating EGF by binding to a similar HRE sequence in our study. Therefore, a regulatory mechanism between HIF1α/HIF2α and EGF exists, and the activation of this pathway promotes the malignant progression of GBM.

## Materials and methods

### Public data collection

Protein expression and correlation, disease-free survival (DFS) and overall survival (OS) were analysed for patients included in TCGA, GTEx and CCLE databases using GEPIA (http://gepia.cancer-pku.cn/detail.php).

### Cell isolation and cell culture

U87MG cells and primary glioblastoma (GBM) cells isolated from tissues after surgery were used in the study, and the detailed methods of sorting GBM cells are presented in the supplementary materials. The tumour tissues obtained from patients were anonymized. U87MG cells were authenticated by STR profiling and all the cells were verified none mycoplasma contamination.

### Clonogenicity and asymmetric division assays

Single cells were plated in 96-well plates and incubated with 1% O_2_ or 21% O_2_ to observe sphere formation at 3, 7, 14 and 21 days. The newly formed spheres were cultured in stem cell medium and differentiation medium to observe asymmetric division at 1, 3 and 5 days.

### Protein detection

Briefly, proteins and mRNAs were detected in GBM tissues and GBM and U87MG cells cultured in 21% O_2_ or 1% O_2_ using immunofluorescence staining, western blotting, RT-qPCR, ELISA and immunohistochemistry and the detailed methods are presented in the supplementary materials.

### Flow cytometry (FCM) analysis

FCM was used to analyse the cell cycle of GBM cells cultured in 21% O_2_ or 1% O_2_. In addition, cells were exposed to TMZ (400 μM) and cultured in 1% O_2_ for another 72 h to detect apoptosis, and the detailed methods are presented in the supplementary materials.

### LDH release assay

Cells at a density of 5 × 10^4^ in a 100-µl suspension were seeded in 96-well plates and cultured in the presence of TMZ in 21% O_2_ or 1% O_2_ for 72 h, and LDH release was detected with a LDH assay kit according to the manufacturer’s instructions. A detailed description of the method is presented in the Supplementary methods.

### CCK-8 assay

GBM and U87MG cells were cultured in 96-well plates in 21% O_2_ or 1% O_2_ for 72 h and in the absence or presence of TMZ (400 μM) for another 48 h to detect cell proliferation. The IC50 values were also calculated by performing CCK-8 assays, and the detailed methods are presented in the supplementary materials.

### Prediction of the HIF1α and HIF2α binding sites in EGF

Hypothetical HIF1α and HIF2α binding sites in EGF promoter were predicted using http://jaspar.genereg.net/. In addition, EGF promoter activity was measured by comparing the luciferase levels. A detailed description of the method used to detect promoter activity is presented in the supplementary materials.

### HIF knockout assays

HIF-knockout (HIF-KO) cells were prepared with HIF1α and HIF2α sgRNAs, and the detailed methods are described in the supplementary materials.

### miRNA-Seq analysis

Control, HIF1α-KO, HIF2α-KO and HIF1α/HIF2α-KO cells were cultured under hypoxic conditions for 24 h and then collected for the miRNA-Seq analysis. A detailed description of the method is presented in the supplementary materials and the results were uploaded in the NCBI Gene Expression Omnibus (GEO) database (www.ncbi.nlm.nih.gov/geo) under accession number GSE142719.

### In vivo experiments

BALB/c-nu mice (male, 4~6 weeks) were used in this study. GBM cells (8 × 10^4^) were injected into the brains of 5 mice, and the animals were fed for 14 days. The tumours and normal tissues were collected to analyse HIF1α and HIF2α expression. HIF-KO cells (8 × 10^4^) were injected into the brains of 200 mice (simple size for each group was estimated by (μ_α_ + μ_β_)^2^× *p*_0_×(1-*p*_0_)/(*p*-*p*_0_)^2^), and the groups included Con, Con+TMZ, HIF1α-KO, HIF1α-KO + TMZ, HIF2α-KO, HIF2α-KO + TMZ, HIF1α/HIF2α-KO, and HIF1α/HIF2α-KO + TMZ. MRI was used to detect tumour volume in five randomly selected mice by SPSS 19.0 on day 21. Tumour tissues were collected from another five mice and protein expression was analysed using IHC, RT-qPCR and western blotting, as described above. The remaining mice were used to record the survival time, and the dead mice were excluded after implantation in three day. The ethics committee of Southwest Hospital at Army Medical University approved all animal procedures.

### Statistical analysis

SPSS 19.0 software was used for statistical analyses. Data are presented as means ± standard deviations (SDs). Student’s *t* test was used to assess the significance of differences between the two groups, and one-way analysis of variance (one-way ANOVA) was performed to compare data from at least three groups. The log-rank test was used to analyse the (Overall Survival) OS or (Disease Free Survival) DFS. Pearson’s correlation coefficients were calculated to analyse the correlations between genes. *P* < 0.05 was considered a statistically significant difference.

## Results

### Effects of HIF1α/HIF2α on the survival time of patients with GBM

**Fig. 1:**
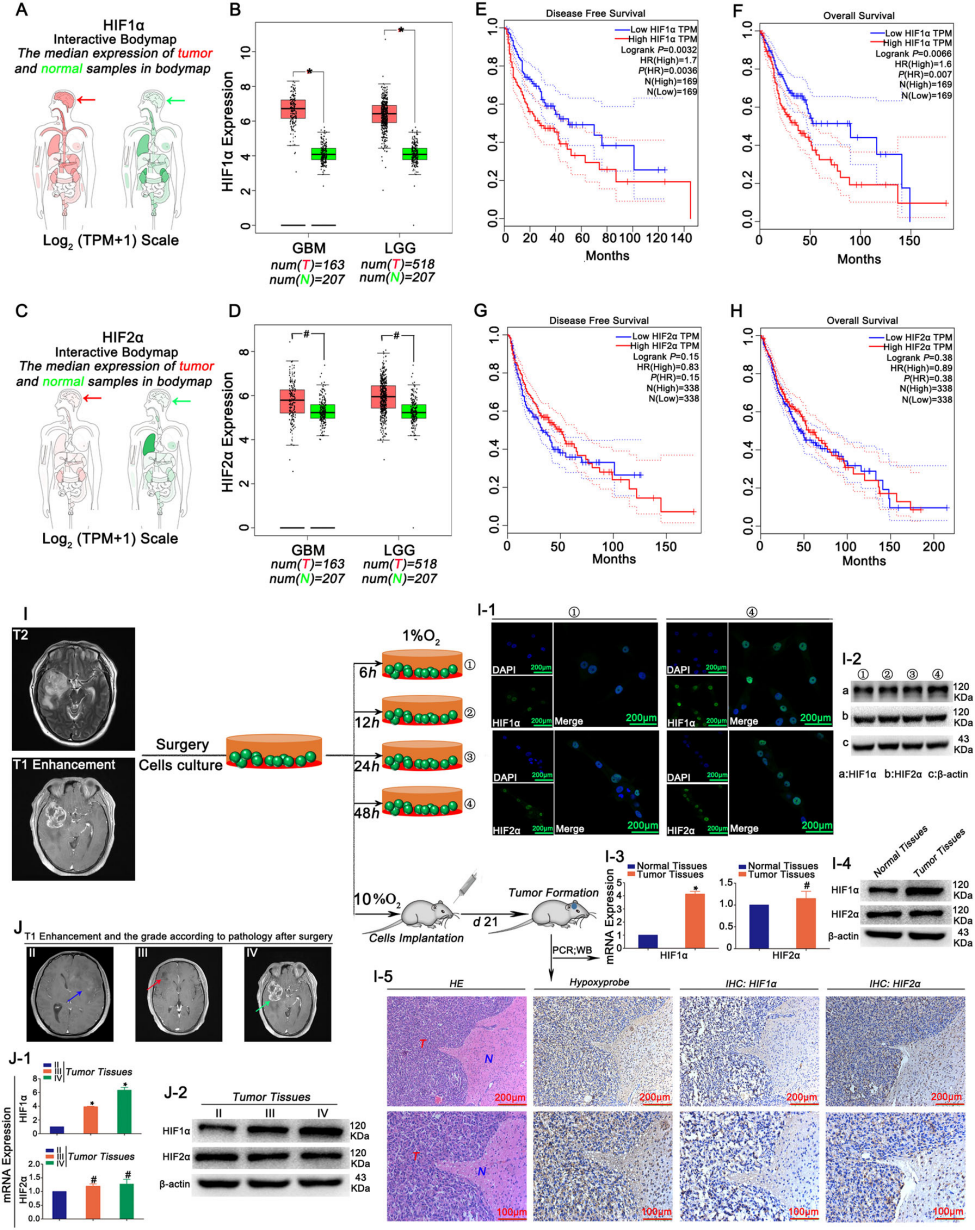
Effects of HIF1α/HIF2α on the survival of patients with GBM. **a**–**d** Both HIF1α and HIF2α were expressed at high levels in GBM; however, only HIF1α showed higher expression in tumours than in normal tissues. **e**–**h** Higher HIF1α expression led to shorter OS and DFS, and no significant differences were observed in OS and DFS between the higher and lower HIF2α expression groups. **i** GBM cells cultured in the presence of 1% O_2_ for 6, 12, 24 and 48 h exhibited increased HIF1α expression, but HIF2α levels were steadily maintained (I.1–I.2). The results revealed higher levels of HIF1α in tumour tissues, but a statistically significant difference in HIF2α levels was not observed between tumour and normal tissues (I.3–I.5). Hypoxyprobe^TM^-1 detection verified the location of glioma in a hypoxic microenvironment (I.5). **j** HIF1α levels increased from WHO II to WHO IV grade tumours, but no significant difference in HIF2α levels was observed. All values are presented as the means ± SD. ^*^P < 0.05 and ^#^*P* > 0.05 were determined using Student’s *t* test or one-way analysis of variance, and the survival time was analysed using the log-rank test.

According to TCGA database, HIF1α was expressed at higher levels in GBM tissues than in normal tissues, but a significant difference in HIF2α expression was not observed between tumour and normal tissues (Fig. 1a–d). In addition, high HIF1α expression led to a shorter DFS and OS, but the DFS and OS of patients were not related to HIF2α expression (Fig. 1e–h). Culturing GBM1 cells in 1% O_2_ for 6, 12, 24 and 48 h increased HIF1α expression. However, no significant difference in HIF2α expression was observed during hypoxia (Fig. 1i and S1A, B). GBM1 cells were implanted into the brains of mice housed in 10% O_2_ for 14 days. HIF1α was expressed at higher levels in tumour tissues than in normal tissues, but a significant difference in HIF2α expression was not observed (Fig. 1i and S1C). In addition, immunohistochemistry verified the effects of the hypoxic microenvironment on GBM tissues (Fig. 1i). Finally, RT-qPCR and western blotting revealed increasing levels of HIF1α in World Health Organization (WHO) grade II glioma to grade IV tumours, but no difference in HIF2α expression was observed (Fig. 1j and S1D).

### Hypoxia promoted arrest in G1 phase and inhibited cell apoptosis

**Fig. 2:**
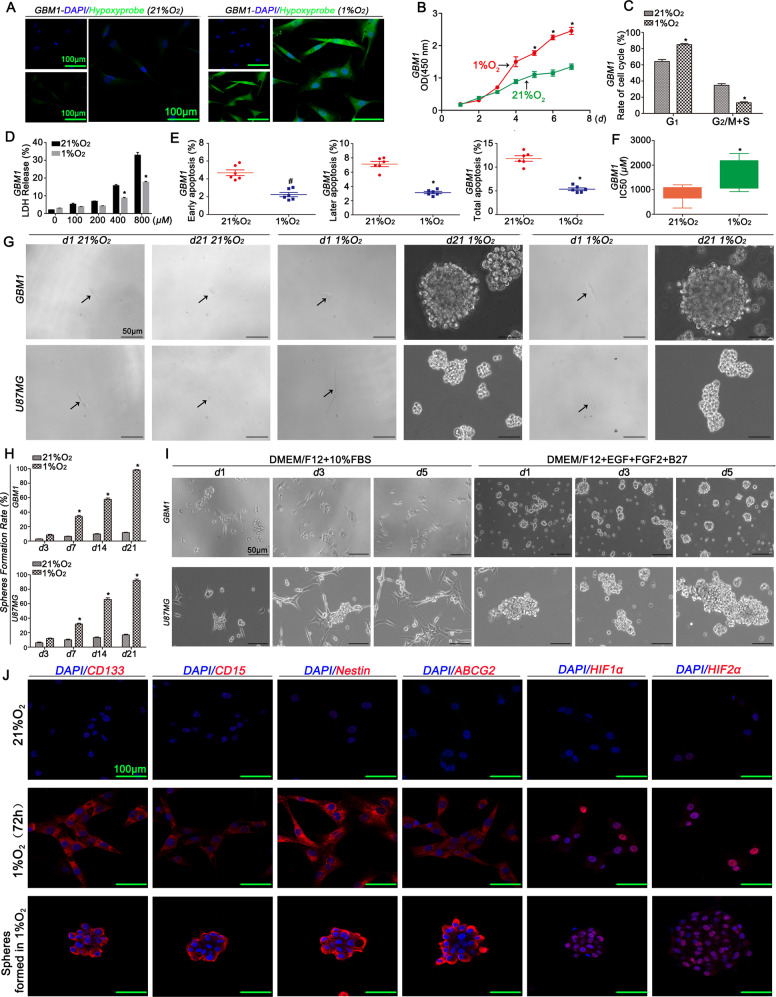
Hypoxia inhibited apoptosis and induced the dedifferentiation of GBM cells. **a** GBM1 cells cultured in the presence of 1% O_2_ presented higher levels of Hypoxyprobe^TM^-1. **b** GBM1 cells cultured in the presence of 1% O_2_ displayed a higher proliferation rate than cells cultured in the presence of 21% O_2_. **c** GBM1 cells exposed to hypoxia for 72 h displayed a higher proportion of cells in G_1_ phase. **d** TMZ (0, 100, 200, 400 and 800 μM) was added to the culture medium of GBM1 cells, and lower levels of LDH release were observed in the hypoxia group than in the control group. **e** TMZ (400 μM) was added to the culture medium of cells cultured in the presence of different concentrations of oxygen for 72 h, and lower percentages of late and total apoptotic cells were observed in the GBM1 cells cultured with 1% O_2_, but no difference was observed in the percentage of early apoptotic cells between the two groups. **f** IC50 values of GBM1 cells cultured under normoxic conditions were lower than cells cultured under hypoxic conditions. **g**–**h** The sphere formation rate of cells cultured in the presence of 1% O_2_ was higher than in cells cultured in the presence of 21% O_2_. **i** Newly formed spheres exhibited asymmetric division. **j** Newly formed spheres and GBM1 cells cultured in the presence of 1% O_2_ for 72 h expressed CD133, CD15, Nestin, ABCG2, HIF1α and HIF2α at high levels, which were not detected in cells cultured under normoxic conditions.^*^P < 0.05 was determined using Student’s *t* test.

Hypoxyprobe^TM^-1 was used to verify that the cells were maintained in the hypoxic microenvironment (Fig. 2a). The hypoxic cells had a higher proliferation rate and a higher proportion of cells in G_1_ phase than the normoxic cells (Fig. 2b, c and S2A). Then, the addition of TMZ (0, 100, 200, 400 and 800 μM) into the medium of GBM cells resulted in lower levels of LDH release under hypoxic conditions (Fig. 2d and S2B). Additionally, the cells exposed to TMZ (400 μM) for 72 h under normoxic conditions were presented higher percentages of later and total apoptosis compared with hypoxic cells (Fig. 2e and S2C). Finally, the IC50 value for GBM1 cells cultured under normoxic conditions was 845.10 ± 423.82 μmol/L, which was much lower than the value for cells cultured under hypoxic conditions (1678.28 ± 586.87 μmol/L, Fig. 2f). A similar significant difference was observed in GBM2 cells (Fig. S2D).

### Hypoxia promoted the dedifferentiation of GBM cells

Morphological changes were observed in only one cell exposed to 21% O_2_ or 1% O_2_, and the cell was dead after exposure to 21% O_2_ for 21 days. However, the cells cultured with 1% O_2_ formed suspended spheres after one week, and the rate of spheres (spheres/*d*3 surviving cells) increased in a time-dependent manner, with a value greater than 95% after exposure for 21 days (Fig. 2g-h). Next, differentiation was assessed in the newly formed spheres cultured with DMEM/F12 + 10% foetal bovine serum (FBS) under a state of growth adherence and the features of stemness were verified after an incubation with DMEM/F12 + EGF + FGF2 + B27 in a state of suspended growth (Fig. 2i). Immunofluorescence staining showed newly formed spheres and the cells cultured in 1% O_2_ for 72 h expressed CD133, CD15, Nestin, ABCG2, HIF1α and HIF2α at high levels (Fig. 2j).

### Simultaneous HIF1α/HIF2α-KO promoted cell proliferation and chemosensitization

**Fig. 3:**
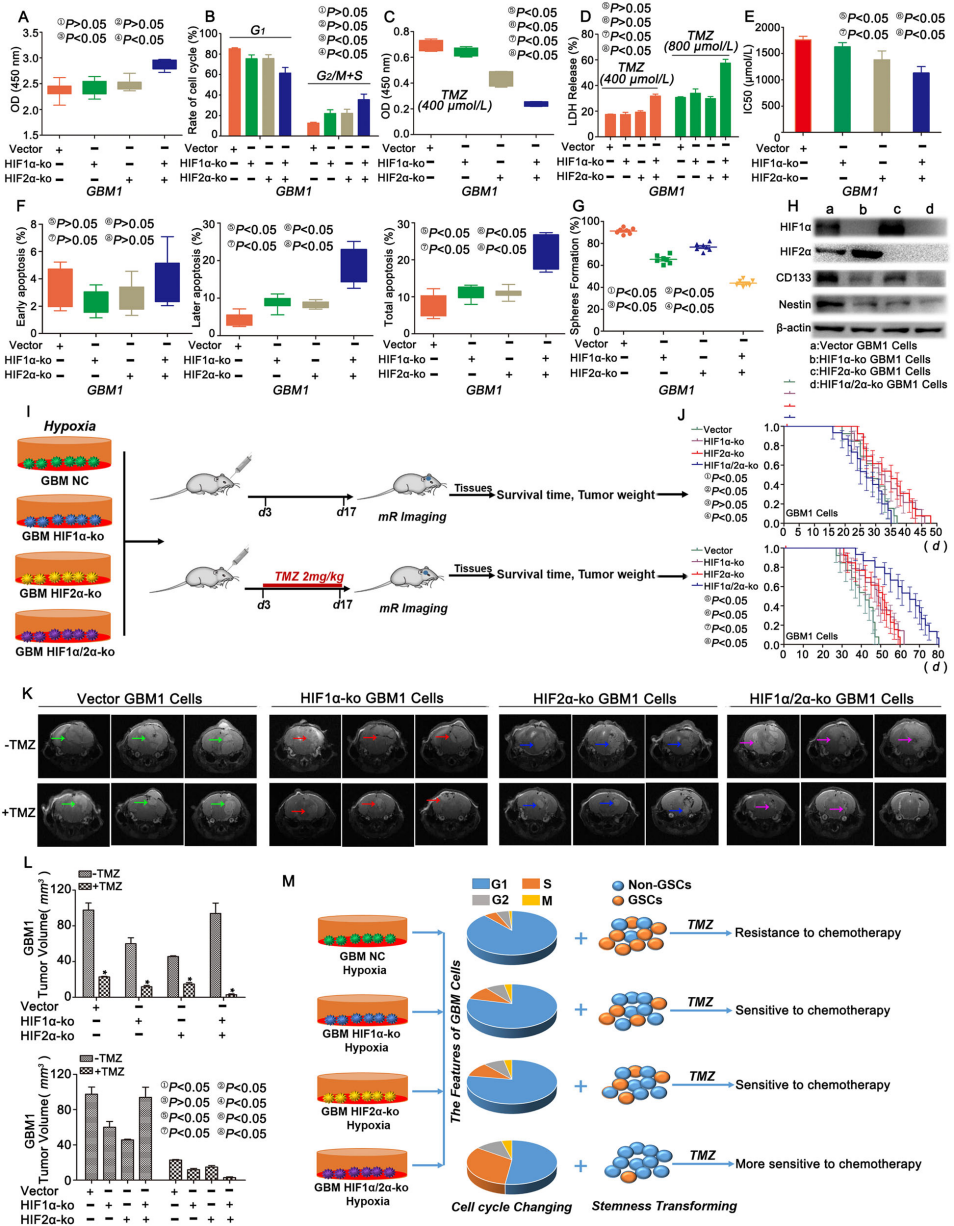
Simultaneous HIF1α and HIF2α knockout increased proliferation and chemosensitization. **a** No significant difference in proliferation was observed in GBM1 cells with single HIF1α or HIF2α knockout, but a higher cell proliferation rate was observed in cells with simultaneous HIF1α and HIF2α knockout in the absence of the TMZ treatment. **b** GBM1 cells with single HIF1α or HIF2α knockout presented no significant differences compared with the control, but a significant difference was observed after simultaneous HIF1α or HIF2α knockout, as this group presented the lowest percentage of cells in G_1_ phase compared with the other groups cultured in the presence of 1% O_2_. **c** HIF1α- or HIF2α-KO GBM1 cells exposed to TMZ (400 μM) for 72 h showed decreased proliferation, and the lowest proliferation rate was observed after simultaneous HIF1α and HIF2α knockout. **d** Higher levels of LDH release were observed in simultaneous HIF1α- and HIF2α-KO cells than in other cells. **e** The IC50 value decreased significantly after HIF1α or HIF2α knockout. **f** The percentages of late and total apoptotic cells increased after HIF1α or HIF2α knockout, and the highest percentage of apoptotic cells was observed in the group with simultaneous HIF1α and HIF2α knockout, but no significant difference was observed in the percentage of early apoptotic cells among groups. **g** A lower sphere formation rate was observed after HIF1α or HIF2α knockout. **h** CD133 and Nestin expression decreased after HIF1α or HIF2α knockout in cells. HIF1α expression increased after HIF2α knockout. In contrast, HIF2α expression increased after HIF1α knockout. **i** Schematic of the in vivo assay. **j**–**l** Analyses of the survival time and tumour volume in control and mice implanted with HIF1α/HIF2α-KO cells and treated with or without TMZ (2 mg/kg). **m** HIF1α or HIF2α knockout alone did not exert significant effects on proliferation and the cell cycle because of substitution effects, but inhibited stemness, leading to chemosensitization after TMZ treatment. However, if HIF1α and HIF2α were knocked out simultaneously, they inhibited cell cycle arrest, promoted proliferation, and decreased stemness, resulting in the chemosensitization of GBM cells. ^*^P < 0.05 was determined using Student’s *t* test, and the specific *P* values are shown in Fig. S5E.

Successful knockout of HIF1α and HIF2α was confirmed by immunofluorescence staining (Fig. S3A). The CCK-8 assay did not reveal significant differences in proliferation among HIF1α-KO cells, HIF2α-KO cells and the control group after culture under hypoxic conditions for 72 h. However, the proliferation rate increased significantly after simultaneous HIF1α and HIF2α knockout (Fig. 3a, S3B and S5E). In addition, single knockout of HIF1α or HIF2α did not affect the number of cells in G_1_ phase, but fewer cells with simultaneous HIF1α and HIF2α knockout were detected in G_1_ phase (Fig. 3b and S5E). Nevertheless, after TMZ exposure, cells with simultaneous HIF1α and HIF2α knockout showed the lowest proliferation rate, the highest level of LDH release, the highest apoptotic rate and the lowest IC50 value (Fig. 3c–f, S3C–F and S5E). The sphere formation rate by a single cell cultured in 1% O_2_ decreased after HIF1α or HIF2α knockout, and the lowest value was observed after simultaneous HIF1α and HIF2α knockout (Fig. 3g, S4A and S5E). In addition, western blots showed an increase in HIF1α levels after HIF2α knockout, an increase in HIF2α levels after HIF1α knockout, and significantly decreased levels of CD133 and Nestin after HIF1α and HIF2α knockout (Fig. 3h, S4B and S5E). These cells were implanted into the brains of the mice, and cells with simultaneous HIF1α and HIF2α knockout produced tumours with a larger volume than control cells, and the tumours of both groups above were larger than groups implanted with single HIF1α or HIF2α knockout cells. An intraperitoneal injection of TMZ (2 mg/kg) into the aforementioned groups reduced the tumour volume in the HIF1α or HIF2α knockout groups, and the smallest tumour volume was observed in the group with dual HIF1α and HIF2α knockout (Fig. 3i. k, l, S5B-C and S5E). The tumour weight showed a similar trend (Fig. S5D, E). Regarding the survival time in the animals without TMZ exposure, HIF1α or HIF2α knockout alone correlated with a longer survival time than the control, but simultaneous HIF1α and HIF2α knockout correlated with a shorter survival time than the control. However, after TMZ exposure, the trend changed; the group with both HIF1α and HIF2α knockout showed the longest survival time compared with the other three groups (Fig. 3j, S5A and S5E).

### HIF1α and HIF2α expression were regulated by miR-210-3p under hypoxic conditions

**Fig. 4:**
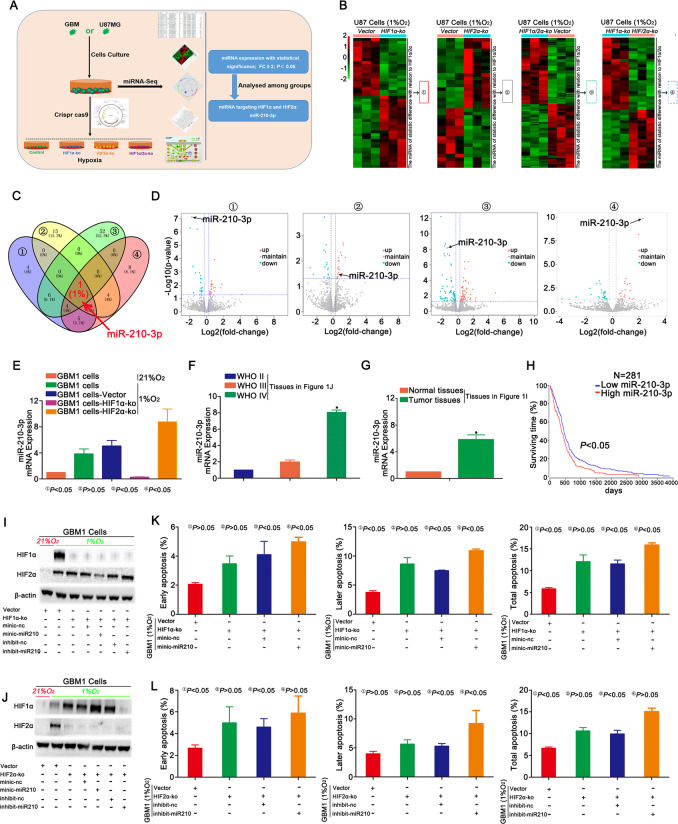
miR-210-3p regulated HIF1α and HIF2α expression in hypoxic cells. **a** Schematic of the mechanistic study. A miRNA-Seq analysis of HIF1α-KO, HIF2α-KO, simultaneous HIF1α- and HIF2α-KO and control cells was performed and revealed statistically significant differences in the expression of miRNAs targeting HIF1α or HIF2α in this process. **b**–**d** Heat maps showed statistically significant changes in the expression of miR-210-3p associated with HIF1α and HIF2α expression. The expression of miR-210-3p decreased in HIF1α-KO cells compared with control cells; however, its expression increased after HIF2α knockout. Compared with the simultaneous HIF1α and HIF2α knockout group, the control group exhibited increased miR-210-3p expression. Finally, for the HIF1α-KO and HIF2α-KO groups, higher miR-210-3p expression was observed in the cells of the HIF2α-KO group. **e** The expression of miR-210-3p was detected in GBM1 cells after HIF1α and HIF2α knockout using RT-qPCR. **f** Higher miR-210-3p expression was observed in WHO grade IV tumours compared with other tumour grades. **g** Higher miR-210-3p expression was observed in tumour tissues than in normal tissues. **h** TCGA database showed a lower survival time in the group with higher miR-210-3p expression. **i**, **j** Changes in HIF1α and HIF2α expression were detected in HIF1α- or HIF2α-KO GBM1 cells overexpressing or silencing for miR-210-3p and cultured in the presence of 1% O_2_. **k**, **l** Apoptosis was detected in HIF1α- or HIF2α-KO GBM1 cells overexpressing or silencing for miR-210-3p expression and cultured in the presence of 1% O_2_. ^*^P < 0.05 was determined using Student’s *t* test, and the specific *P* values are shown in Fig. S6D.

HIF1α-KO, HIF2α-KO, dual HIF1α and HIF2α knockout and control cells were subjected to miRNA-Seq analysis to identify miRNAs that target HIF1α or HIF2α (Fig. 4a). The results showed that only miR-210-3p was the common miRNA identified in the groups and presented a statistically significant relationship with HIF1α and HIF2α expression (Fig. 4b, c and S6A and Supplementary Table S1). The heat map and volcano plot revealed a decrease in miR-210-3p expression in HIF1α-KO cells and an increase in its expression after HIF2α knockout. Next, we compared the group in which HIF1α and HIF2α were knocked out simultaneously with the control and found control group expressed miR-210-3p at higher levels. Finally, the HIF1α-KO and HIF2α-KO groups were compared, and higher miR-210-3p expression was observed in the HIF2α-KO group (Fig. 4b–d). RT-qPCR data verified this significant result (Fig. 4e and S6D). Glioma tissues were also detected, and the WHO IV group presented a higher level of miR-210-3p than the other groups (Fig. 4f), and the expression was much higher in tumour tissues than in normal tissues (Fig. 4g). Finally, according to TCGA database, the survival time was lower for patients with tumours displaying higher miR-210-3p expression (Fig. 4h). Next, the relationship between HIF1α, HIF2α and miR-210-3p was examined. First, HIF1α-KO or HIF2α-KO GBM cells were cultured in 1% O_2_, and miR-210-3p was overexpressed or inhibited. HIF2α levels decreased with miR-210-3p overexpression and increased when miR-210-3p expression was inhibited (Fig. 4i and S7). HIF1α expression increased with miR-210-3p overexpression and decreased when miR-210-3p expression was inhibited (Fig. 4j and S7). Finally, the cell apoptosis assay showed that miR-210-3p overexpression in HIF1α-KO cells or miR-210-3p silencing in HIF2α-KO cells led to a higher apoptotic rate (Fig. 4k-l and S6B-D).

### HIF1α and HIF2α regulated the malignant progression of GBM through EGF

**Fig. 5:**
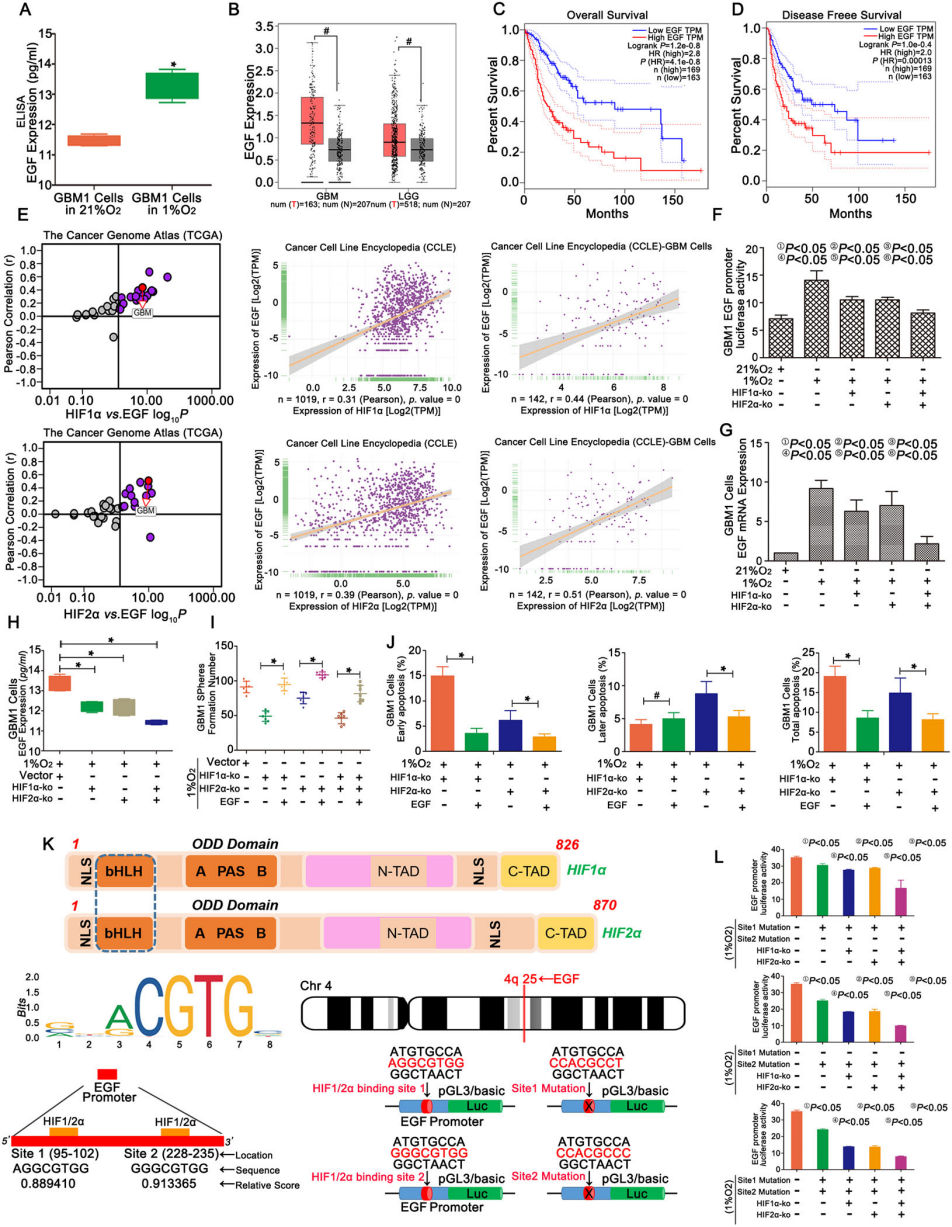
HIF1α and HIF2α regulated GBM growth and apoptosis through EGF. **a** An ELISA showed higher levels of EGF in GBM1 cells after culture with 1% O_2_ for 72 h. **b** EGF was expressed at high levels in tumours, but the difference between the tumour and normal tissues was not significant. **c**, **d** Longer OS and DFS were observed in the group with lower EGF expression than in the control group. **e** A positive correlation was observed between HIF1α, HIF2α and EGF expression, according to TCGA and CCLE databases. **f**–**h** Decreases in the luciferase activity of the EGF promoter and levels of the EGF mRNA and protein were observed in single HIF1α- or HIF2α-KO cells compared with the control. However, all of these groups showed higher levels than simultaneous HIF1α- and HIF2α-KO cells. **i** The number of spheres increased after EGF was added to the culture medium of HIF1α- or HIF2α-KO cells cultured in the presence of 1% O_2_. **j** The percentage of apoptotic in HIF1α- or HIF2α-KO cells decreased after the addition of EGF to the culture medium. **k** Bioinformatics analysis of the HREs of EGF based on EGF family binding sites. Two predicted binding regions were shared: one sequence was 5′-AGGCGTGG-3′ and the other was 5′-GGGCGTGG-3′. In addition, the two predicted sequences were mutated. **l** Detection of the luciferase activity of the EGF promoter after the mutation of the two predicted sequences in control cells, HIF1α- or HIF2α-KO cells cultured in the presence of 1% O_2_. ^*^P < 0.05 and ^#^*P* > 0.05 were determined using Student’s *t* test or one-way analysis of variance, and the specific *P* values are shown in Fig. S8I.

ELISA and immunofluorescence staining showed higher levels of EGF after cells were cultured in 1% O_2_ for 72 h than in the normoxic cells, and the spheres formed in 1% O_2_ showed higher expression of EGF (Fig. 5a and S8A-B). Moreover, TCGA database showed high expression of EGF in GBM (Fig. 5b). The OS and DFS of patients with low EGF levels were much longer than patients with high EGF levels (Fig. 5c-d). TCGA and CCLE data showed that both HIF1α and HIF2α positively regulated EGF expression (Fig. 5e). The EGF promoter activity in the hypoxic group was higher than in the normoxic group, and lower EGF promoter activity was observed in the cells with single HIF1α or HIF2α knockout compared with the control cells. All the cells listed above exhibited higher EGF promoter activity than cells with dual HIF1α and HIF2α knockout (Fig. 5f, S8C and S8I). A similar trend was observed for mRNA and protein levels (Fig. 5g-h, S8D-E and S8I). In addition, the changes in sphere formation were recorded after the addition of EGF to the culture medium of HIF1α- or HIF2α-KO cells, resulting an increase in the number of spheres (Fig. 5i and S8F-G). Finally, FCM revealed a decreased apoptotic rate after the addition of EGF to the culture medium of all groups (Fig. 5j and S8H). A bioinformatics analysis was performed to identify the hypoxia-response elements (HREs) in the EGF sequence, and two predicted binding regions were found: one sequence was 5′-AGGCGTGG-3′ with a relative score of 0.889410 (site 1) and the other was 5′-GGGCGTGG-3′ with a relative score of 0.913365 (site 2). The two predicted sequences were mutated and the change in luciferase activity in HIF1α- or HIF2α-KO cells was detected to verify the regulatory process (Fig. 5k). For the control cells without HIF1α or HIF2α knockout, the luciferase activity decreased after each of the two predicted sequences was mutated and reached the lowest level in the group in which both sequences were mutated. For cells carrying site 1 or site 2 mutations alone, the results revealed decreased luciferase activity after HIF1α or HIF2α knockout, and the cells with simultaneous HIF1α and HIF2α knockout showed the lowest luciferase activity among groups. Then, the changes in the luciferase activity were analysed for cells after dual site 1 and site 2 mutations, and among all groups, the lowest luciferase activity was observed when HIF1α and HIF2α were simultaneously deleted (Fig. 5l and S8I).

## Discussion

GBM is observed in the hypoxic microenvironment^22,23^, which is mainly regulated by HIF1α and HIF2α^5,7,24^. Previous studies explored the long-term contributions of these proteins to tumour growth and revealed an important role for HIF1α^2,8,25^. HIF2α primarily functions in glioma stem cells (GSCs) and promotes GSCs radiochemoresistance by maintaining stemness under hypoxia conditions^5,26^. Therefore, the inhibition of HIF1α or HIF2α inhibits the malignant progression of GBM cells^8,25^. For example, the inhibition of HIF1α increases the sensitivity of GBM cells to chemotherapeutic drugs^27^ and a strategy targeting HIF2α in GSCs attenuated the tumour initiation potential^5,28^. Therefore, these studies strongly support the necessary development of pharmacological HIF inhibitors as treatments for GBM, as they will theoretically inhibit tumour progression. As a result, several HIF inhibitors have been approved in phase trials^29^. However, until now no HIF-targeted therapies have cured patients successfully with GBM.

HIF1α or HIF2α was deleted to observe dedifferentiation, proliferation and chemoresistance in order to explore the failure of inhibitors targeting these proteins. Regarding dedifferentiation, previous studies have confirmed that GSCs develop from non-GSCs in response to therapeutic stress, such as TMZ^30,31^ and ionizing radiation^32^. As a result, studies have proposed the hypothesis that hypoxia may also induce dedifferentiation^33–35^; however, none of the studies have conclusively verified this hypothesis. Therefore, we detected dedifferentiation and found more than 95% of single GBM cells formed spheres after culture under hypoxic conditions for 21 days, while very few spheres formed under normoxic conditions. Even if 20% of the newly formed spheres were derived from GSCs existing in GBM itself (the percentage of GSCs in GBM is approximately 20%^36^), the other 75% of the newly formed spheres should be derived from differentiated non-GSCs after dedifferentiation. Our results confirming this mechanism were consistent with previous studies showing that stemness decreased after HIF1α or HIF2α knockout^2,5,28^. Nevertheless, unlike previous studies^37,38^, no significant differences in proliferation and the cell cycle were observed in vitro after single HIF1α or HIF2α knockout, which may be due to the shorter detection time than used in previous studies. However, HIF1α or HIF2α knockout alone inhibited tumour growth in vivo. Furthermore, we emphasized that the proliferation rate was accelerated after simultaneous HIF1α and HIF2α knockout. This result revealed a substantial difference from the theory that simultaneous HIF1α and HIF2α knockout would result in the lowest proliferation rate. Surprisingly, the opposite result was observed after exposure to TMZ, indicating that simultaneous HIF1α and HIF2α knockout cells became chemosensitized. This study is significance, as GBM was successfully cured by simultaneously targeting both HIF1α and HIF2α and administering TMZ.

**Fig. 6:**
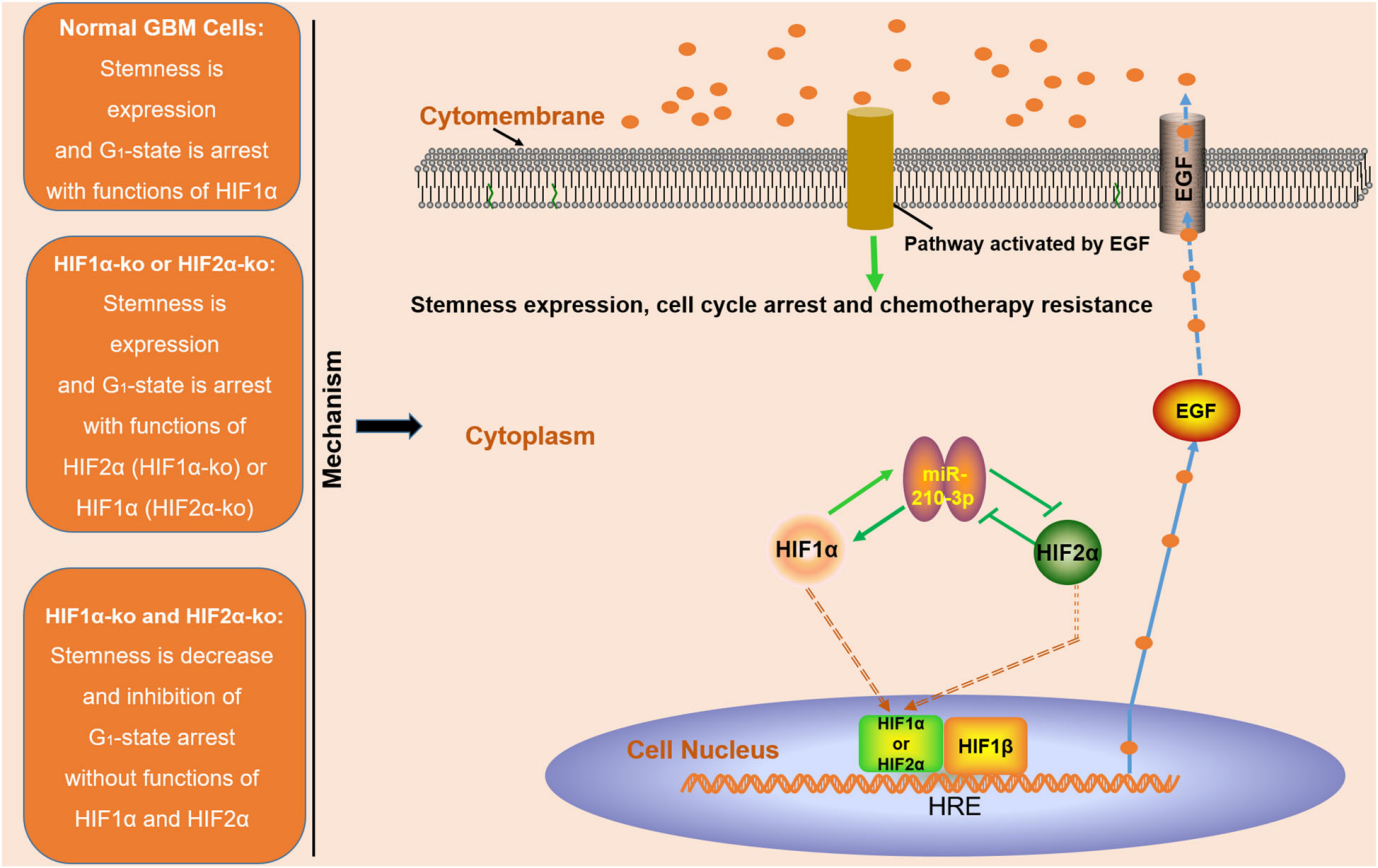
The novel mechanism by which HIF1α and HIF2α regulate the malignant progression of GBM through miR-210-3p and EGF. HIF1α and HIF2α mutually regulate each other through a negative feedback loop mediated by miR-210-3p, and high levels of miR-210-3p lead to higher HIF1α expression. HIF1α and HIF2α are upstream regulators of the transcription of the EGF gene, which regulates GBM malignancy through the related signalling pathway activated by EGF. In summary, HIF1α/HIF2α-miR-210-3p are critical factors that contribute to GBM growth, dedifferentiation and chemoresistance by regulating pathways activated by EGF in hypoxic cells, thus increasing GBM malignancy.

Next, we wondered why HIF1α or HIF2α knockout alone was unable to substantially decrease the tumour volume. Interestingly, the expression of HIF2α increased after HIF1α knockout and HIF1α expression increased after HIF2α knockout. Thus, another HIFα factor promotes tumour growth after one subunit is knocked out, leading to an unremarkable decrease in tumour volume. Consistent with previous studies, stemness was decreased after single HIF1α or HIF2α knockout, explaining why the knockout of either HIF1α or HIF2α resulted in chemosensitization. Nevertheless, simultaneously knockout of HIF1α and HIF2α not only promoted cell cycle progression without any substitution effects but also decreased the stemness more noticeably, remarkably increasing proliferation and chemosensitization (Fig. 3m). The mechanism by which HIF1α and HIF2α regulate each other remains unknown, and thus hypoxia-related miRNAs were the focus of this study. Both previous studies and our study indicated that a positive feedback loop existed between HIF1α and miR-210-3p^17,39^; however, no studies have elucidated the regulatory mechanism between HIF2α and miR-210-3p. By performing experiments, we confirmed that high levels of HIF1α increased miR-210-3p expression in hypoxic cells, restricting the expression of HIF2α. However, if HIF1α was knocked out, miR-210-3p expression decreased, thus HIF2α expression increased (Fig. 6). This new regulatory mechanism explains the relationship between HIF1α, HIF2α and miR-210-3p, potentially representing a new target for GBM treatment.

EGF, an upstream protein of HIF1α, has been reported in glioma^29,39^. Unexpectedly, in the present study, EGF expression decreased after HIF1α and HIF2α knockout. Actually, the two HIFα proteins are highly homologous and regulate some similar genes, suggesting that they may bind to similar HRE sequences^40,41^. Both HIF1α and HIF2α contain a conserved DNA binding region known as the bHLH-PAS domain, which is a common domain shared by HIF1α and HIF2α that binds to HREs and induces a series of responses^8^. Therefore, by performing a series of experiments, both HIF1α and HIF2α were verified as upstream genes that regulate EGF by binding the DNA sequences AGGCGTGG and GGGCGTGG in GBM cells. Therefore, feedback regulation exists in hypoxic cells between HIF1α, HIF2α and EGF. EGF contributes to HIF1α expression; in contrast, upregulation of HIF1α and HIF2α promoting EGF expression (Fig. 6).

In summary, researchers should create a better treatment to improve the prognosis of patients with GBM. Based on our findings, HIF1α/HIF2α-miR-210-3p regulates the malignant progression of GBM through EGF, which provides a new target strategy for GBM treatment.

## Supplementary information

Supplementary table 1

Supplementary table 2

Supplementary table 3

Supplementary table 4

Supplementary table 5

Supplementary table 6

Supplementary Figure 1

Supplementary Figure 2

Supplementary Figure 3

Supplementary Figure 4

Supplementary Figure 5

Supplementary Figure 6

Supplementary Figure 7

Supplementary Figure 8

Supplementary_Figure_Legends

Supplementary_materials_and_methods
