# Supplementary material for: The HIF1α/HIF2α-miR210-3p network regulates glioblastoma cell proliferation, dedifferentiation and chemoresistance through EGF under hypoxic conditions
Source: Cell Death Dis. 2020 Nov 18;11(11):992. doi: 10.1038/s41419-020-03150-0 (PMC7674439; doi:10.1038/s41419-020-03150-0)
Supplement: Supplementary file 1 — Supplementary table 1 [file 41419_2020_3150_MOESM1_ESM.docx]

Table S1 The miRNAs showed differences in expression among groups

| **U87 *VS* U87 HIF1α-ko cells** |  | **U87 *VS* U87 HIF2α-ko cells** |  |
| --- | --- | --- | --- |
| miRNA | diffState | miRNA | diffState |
| hsa-miR-490-5p | up | hsa-miR-652-5p | up |
| hsa-miR-4423-3p | up | hsa-miR-19b-3p | up |
| hsa-miR-6505-5p | up | hsa-miR-10395-5p | up |
| hsa-miR-582-3p | up | hsa-miR-887-3p | up |
| hsa-miR-19b-3p | up | hsa-miR-20b-5p | up |
| hsa-miR-616-5p | up | hsa-miR-671-5p | up |
| hsa-miR-19a-3p | up | hsa-miR-12136 | up |
| hsa-miR-1301-3p | up | hsa-miR-99a-3p | up |
| hsa-miR-378d | up | hsa-miR-17-5p | up |
| hsa-miR-146b-5p | up | hsa-miR-34c-5p | up |
| hsa-miR-192-5p | up | hsa-miR-424-5p | up |
| hsa-miR-362-5p | up | hsa-miR-20a-5p | up |
| hsa-miR-378c | up | hsa-miR-138-5p | up |
| hsa-miR-3681-5p | up | hsa-miR-182-5p | up |
| hsa-miR-660-5p | up | hsa-miR-146b-5p | up |
| hsa-miR-378a-3p | up | hsa-miR-210-3p | up |
| hsa-miR-146a-5p | up | hsa-miR-16-5p | up |
| hsa-miR-182-5p | up | hsa-miR-146a-5p | up |
| hsa-miR-96-5p | up | hsa-miR-93-5p | up |
| hsa-miR-215-5p | up | hsa-miR-21-5p | up |
| hsa-miR-204-5p | up | hsa-let-7d-5p | down |
| hsa-miR-17-5p | up | hsa-miR-27b-5p | down |
| hsa-miR-532-5p | up | hsa-miR-218-5p | down |
| hsa-miR-320a-3p | up | hsa-miR-128-3p | down |
| hsa-miR-320b | up | hsa-miR-3074-3p | down |
| hsa-miR-615-3p | down | hsa-miR-2355-3p | down |
| hsa-miR-9985 | down | hsa-miR-654-3p | down |
| hsa-miR-7-1-3p | down | hsa-miR-615-3p | down |
| hsa-let-7b-3p | down | hsa-miR-3617-5p | down |
| hsa-miR-873-3p | down | hsa-miR-379-5p | down |
| hsa-miR-424-3p | down | hsa-miR-5100 | down |
| hsa-miR-4443 | down | hsa-miR-381-3p | down |
| hsa-miR-196a-5p | down | hsa-miR-411-3p | down |
| hsa-let-7i-3p | down | hsa-let-7f-1-3p | down |
| hsa-miR-767-5p | down | hsa-miR-299-5p | down |
| hsa-miR-7974 | down | hsa-miR-1297 | down |
| hsa-miR-503-5p | down | hsa-miR-485-5p | down |
| hsa-miR-550a-3p | down |  |  |
| hsa-miR-584-5p | down |  |  |
| hsa-miR-105-5p | down |  |  |
| hsa-miR-767-3p | down |  |  |
| hsa-miR-218-2-3p | down |  |  |
| hsa-miR-105-3p | down |  |  |
| hsa-miR-210-3p | down |  |  |
| hsa-miR-210-5p | down |  |  |

| **U87 *VS* U87 HIF1α/2α-ko cells** | | **U87 HIF1α-ko *VS* U87 HIF2α-ko cells** | |
| --- | --- | --- | --- |
| miRNA | diffState | miRNA | diffState |
| hsa-miR-466 | up | hsa-miR-210-3p | up |
| hsa-miR-561-5p | up | hsa-miR-210-5p | up |
| hsa-miR-326 | up | hsa-miR-876-5p | up |
| hsa-miR-4423-5p | up | hsa-miR-105-3p | up |
| hsa-miR-362-3p | up | hsa-miR-10395-5p | up |
| hsa-miR-4423-3p | up | hsa-miR-2277-3p | up |
| hsa-miR-6505-5p | up | hsa-miR-940 | up |
| hsa-miR-652-5p | up | hsa-miR-671-5p | up |
| hsa-miR-26a-2-3p | up | hsa-miR-193b-3p | up |
| hsa-miR-190a-5p | up | hsa-miR-550a-3p | up |
| hsa-miR-502-5p | up | hsa-miR-424-5p | up |
| hsa-miR-582-3p | up | hsa-miR-584-5p | up |
| hsa-miR-5010-3p | up | hsa-miR-6134 | up |
| hsa-miR-365a-5p | up | hsa-miR-877-5p | up |
| hsa-miR-365a-3p | up | hsa-miR-4787-3p | up |
| hsa-miR-365b-3p | up | hsa-miR-7974 | up |
| hsa-miR-616-5p | up | hsa-miR-503-5p | up |
| hsa-miR-29b-3p | up | hsa-miR-873-3p | up |
| hsa-miR-582-5p | up | hsa-miR-105-5p | up |
| hsa-miR-29a-5p | up | hsa-miR-767-5p | up |
| hsa-miR-34a-3p | up | hsa-miR-138-5p | up |
| hsa-miR-500b-5p | up | hsa-miR-92b-3p | up |
| hsa-miR-500a-5p | up | hsa-miR-378a-3p | down |
| hsa-miR-30a-5p | up | hsa-miR-505-3p | down |
| hsa-miR-18a-5p | up | hsa-miR-24-2-5p | down |
| hsa-miR-605-5p | up | hsa-miR-192-5p | down |
| hsa-miR-30c-2-3p | up | hsa-miR-215-5p | down |
| hsa-miR-18b-5p | up | hsa-miR-378c | down |
| hsa-miR-19a-3p | up | hsa-miR-204-5p | down |
| hsa-miR-19b-3p | up | hsa-miR-211-5p | down |
| hsa-miR-130a-5p | up | hsa-miR-378d | down |
| hsa-miR-204-5p | up | hsa-miR-494-3p | down |
| hsa-miR-501-5p | up | hsa-miR-128-3p | down |
| hsa-miR-129-1-3p | up | hsa-miR-3909 | down |
| hsa-miR-181b-3p | up | hsa-miR-9903 | down |
| hsa-miR-129-2-3p | up | hsa-miR-7706 | down |
| hsa-miR-877-5p | up | hsa-miR-2355-3p | down |
| hsa-miR-182-5p | up | hsa-miR-379-5p | down |
| hsa-miR-664a-3p | up | hsa-miR-3074-3p | down |
| hsa-miR-29b-1-5p | up | hsa-miR-654-3p | down |
| hsa-miR-660-5p | up | hsa-miR-3617-5p | down |
| hsa-miR-10a-3p | up | hsa-miR-381-3p | down |
| hsa-miR-20a-5p | up | hsa-miR-505-5p | down |
| hsa-miR-101-3p | up | hsa-miR-758-3p | down |
| hsa-miR-96-5p | up | hsa-miR-582-3p | down |
| hsa-miR-211-5p | up | hsa-miR-543 | down |
| hsa-miR-454-3p | up | hsa-miR-378i | down |
| hsa-miR-148b-5p | up | hsa-miR-1297 | down |
| hsa-miR-378f | up | hsa-miR-485-5p | down |
| hsa-miR-362-5p | up |  |  |
| hsa-miR-192-5p | up |  |  |
| hsa-miR-149-5p | up |  |  |
| hsa-miR-17-5p | up |  |  |
| hsa-miR-146b-5p | up |  |  |
| hsa-miR-548k | up |  |  |
| hsa-miR-342-3p | up |  |  |
| hsa-miR-1293 | up |  |  |
| hsa-miR-215-5p | up |  |  |
| hsa-miR-378a-3p | up |  |  |
| hsa-miR-378c | up |  |  |
| hsa-miR-30e-5p | up |  |  |
| hsa-miR-139-5p | up |  |  |
| hsa-miR-21-5p | up |  |  |
| hsa-miR-29a-3p | up |  |  |
| hsa-miR-16-5p | up |  |  |
| hsa-miR-15b-5p | down |  |  |
| hsa-miR-25-3p | down |  |  |
| hsa-let-7a-5p | down |  |  |
| hsa-let-7c-5p | down |  |  |
| hsa-let-7g-5p | down |  |  |
| hsa-miR-671-3p | down |  |  |
| hsa-let-7b-5p | down |  |  |
| hsa-miR-1307-3p | down |  |  |
| hsa-let-7i-5p | down |  |  |
| hsa-miR-92b-5p | down |  |  |
| hsa-miR-503-5p | down |  |  |
| hsa-miR-106b-3p | down |  |  |
| hsa-miR-455-3p | down |  |  |
| hsa-let-7d-3p | down |  |  |
| hsa-miR-130b-5p | down |  |  |
| hsa-miR-23b-5p | down |  |  |
| hsa-miR-200c-3p | down |  |  |
| hsa-miR-550a-3-5p | down |  |  |
| hsa-miR-218-5p | down |  |  |
| hsa-miR-195-3p | down |  |  |
| hsa-miR-1908-5p | down |  |  |
| hsa-miR-193a-5p | down |  |  |
| hsa-let-7d-5p | down |  |  |
| hsa-miR-3661 | down |  |  |
| hsa-miR-335-5p | down |  |  |
| hsa-miR-449c-5p | down |  |  |
| hsa-miR-767-5p | down |  |  |
| hsa-miR-105-5p | down |  |  |
| hsa-miR-3681-5p | down |  |  |
| hsa-miR-196a-5p | down |  |  |
| hsa-miR-424-3p | down |  |  |
| hsa-miR-3617-5p | down |  |  |
| hsa-miR-3182 | down |  |  |
| hsa-miR-4443 | down |  |  |
| hsa-let-7i-3p | down |  |  |
| hsa-miR-449a | down |  |  |
| hsa-miR-2277-5p | down |  |  |
| hsa-miR-381-3p | down |  |  |
| hsa-miR-1185-2-3p | down |  |  |
| hsa-miR-615-3p | down |  |  |
| hsa-miR-1185-1-3p | down |  |  |
| hsa-miR-134-5p | down |  |  |
| hsa-miR-758-3p | down |  |  |
| hsa-miR-411-5p | down |  |  |
| hsa-miR-4324 | down |  |  |
| hsa-miR-654-5p | down |  |  |
| hsa-miR-382-5p | down |  |  |
| hsa-miR-335-3p | down |  |  |
| hsa-miR-382-3p | down |  |  |
| hsa-miR-379-5p | down |  |  |
| hsa-miR-210-5p | down |  |  |
| hsa-miR-409-5p | down |  |  |
| hsa-miR-218-2-3p | down |  |  |
| hsa-miR-654-3p | down |  |  |
| hsa-miR-411-3p | down |  |  |
| hsa-miR-495-3p | down |  |  |
| hsa-miR-409-3p | down |  |  |
| hsa-miR-323a-3p | down |  |  |
| hsa-miR-4636 | down |  |  |
| hsa-miR-210-3p | down |  |  |
| hsa-miR-329-3p | down |  |  |
| hsa-miR-379-3p | down |  |  |
| hsa-miR-299-3p | down |  |  |
