# Supplementary material for: The HIF1α/HIF2α-miR210-3p network regulates glioblastoma cell proliferation, dedifferentiation and chemoresistance through EGF under hypoxic conditions
Source: Cell Death Dis. 2020 Nov 18;11(11):992. doi: 10.1038/s41419-020-03150-0 (PMC7674439; doi:10.1038/s41419-020-03150-0)
Supplement: Supplementary file 2 — Supplementary table 2 [file 41419_2020_3150_MOESM2_ESM.docx]

Table S2 The sequences of primers used for RT-qPCR detection

| EGF | Forward(5'-3') | GCCCCCCTGCCTCCTCCAAGTG |
| --- | --- | --- |
|  | Reverse(5'-3') | GGGGGTGGAGTAGAGTCAGGGCAA |
| HIF1A | Forward(5'-3') | ACTGCACAGGCCACATTCACG |
|  | Reverse(5'-3') | AATCAGCACCAAGCAGGTCATAGG |
| HIF2A | Forward(5'-3') | GCGACCATGAGGAGATTCGTGAG |
|  | Reverse(5'-3') | CAGGTGGCTGACTTGAGGTTGAC |
| β-Actin | Forward(5'-3') | ACCCGCCGCCAGCTCACC |
|  | Reverse(5'-3') | GGGGGGCACGAAGGCTCATC |
| MiRNA-210-3p | Forward(5'-3') | CTGTGCGTGTGACAGCGG |
