# Supplementary material for: The HIF1α/HIF2α-miR210-3p network regulates glioblastoma cell proliferation, dedifferentiation and chemoresistance through EGF under hypoxic conditions
Source: Cell Death Dis. 2020 Nov 18;11(11):992. doi: 10.1038/s41419-020-03150-0 (PMC7674439; doi:10.1038/s41419-020-03150-0)
Supplement: Supplementary file 3 — Supplementary table 3 [file 41419_2020_3150_MOESM3_ESM.docx]

Table S3 The sequences of the miR-210-3p mimic and inhibitor

| NC mimics | Sense(5'-3') | UUCUCCGAACGUGUCACGUTT |
| --- | --- | --- |
|  | Antisense(5'-3') | ACGUGACACGUUCGGAGAATT |
| hsa-miR-210-3p mimics | Sense(5'-3') | CUGUGCGUGUGACAGCGGCUGA |
|  | Antisense(5'-3') | AGCCGCUGUCACACGCACAGUU |
| NC inhibitor | Sense(5'-3') | CAGUACUUUUGUGUAGUACAA |
| hsa-miR-210-3p inhibitor | Sense(5'-3') | UCAGCCGCUGUCACACGCACAG |
