# Supplementary material for: The HIF1α/HIF2α-miR210-3p network regulates glioblastoma cell proliferation, dedifferentiation and chemoresistance through EGF under hypoxic conditions
Source: Cell Death Dis. 2020 Nov 18;11(11):992. doi: 10.1038/s41419-020-03150-0 (PMC7674439; doi:10.1038/s41419-020-03150-0)
Supplement: Supplementary file 4 — Supplementary table 4 [file 41419_2020_3150_MOESM4_ESM.docx]

Table S4 The sequences of sgRNAs used to knockout HIF1α and HIF2α

| Target | Oligonucleotide sequence(5'-3') |
| --- | --- |
| HIF1A | GAACTCACATTATGTGGAAG |
| HIF2A | CTTGGAGGGTTTCATTGCCG |
