# Supplementary material for: The HIF1α/HIF2α-miR210-3p network regulates glioblastoma cell proliferation, dedifferentiation and chemoresistance through EGF under hypoxic conditions
Source: Cell Death Dis. 2020 Nov 18;11(11):992. doi: 10.1038/s41419-020-03150-0 (PMC7674439; doi:10.1038/s41419-020-03150-0)
Supplement: Supplementary file 6 — Supplementary table 6 [file 41419_2020_3150_MOESM6_ESM.docx]

Table S6 Primary antibodies used for immunofluorescence or immunohistochemistry detection

| Antigens | Manufacturer | Catalogue numbers | Application |
| --- | --- | --- | --- |
| HIF1A | abcam | ab179483 | 1:100 |
| HIF2A | abcam | ab207607 | 1:100 |
| CD15 | NOVUS | NB100-1831 | 1:100 |
| CD133 | NOVUS | NB120-16518 | 1:100 |
| NESTIN | CST | 33475S | 1:100 |
| ABCG2 | abcam | ab207732 | 1:100 |
| EGF | abcam | ab9695 | 1:100 |
