# Supplementary figures and images for: The HIF1α/HIF2α-miR210-3p network regulates glioblastoma cell proliferation, dedifferentiation and chemoresistance through EGF under hypoxic conditions
Source: Cell Death Dis. 2020 Nov 18;11(11):992. doi: 10.1038/s41419-020-03150-0 (PMC7674439; doi:10.1038/s41419-020-03150-0)

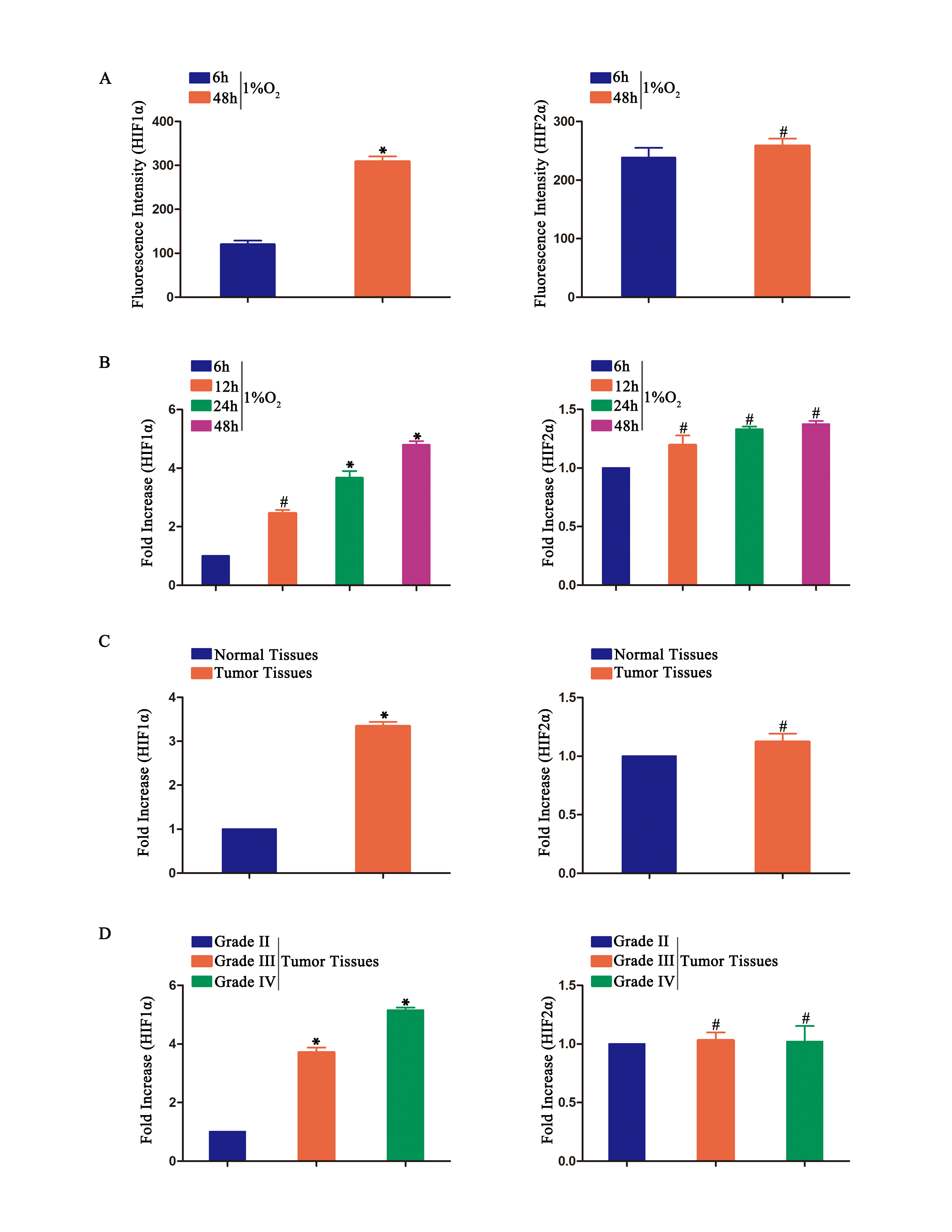

Supplement: Supplementary file 7 — Supplementary Figure 1 [file 41419_2020_3150_MOESM7_ESM.tif]

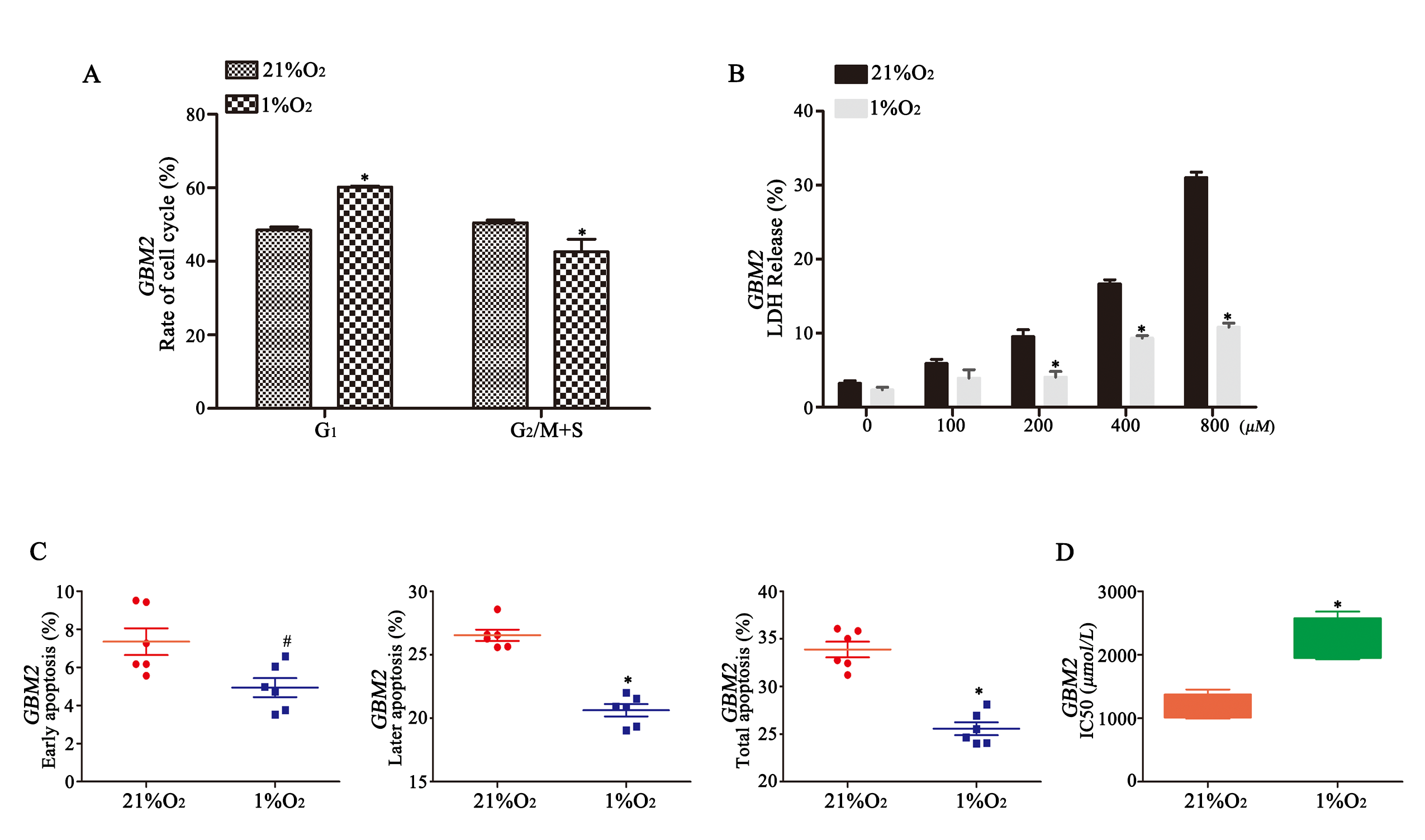

Supplement: Supplementary file 8 — Supplementary Figure 2 [file 41419_2020_3150_MOESM8_ESM.tif]

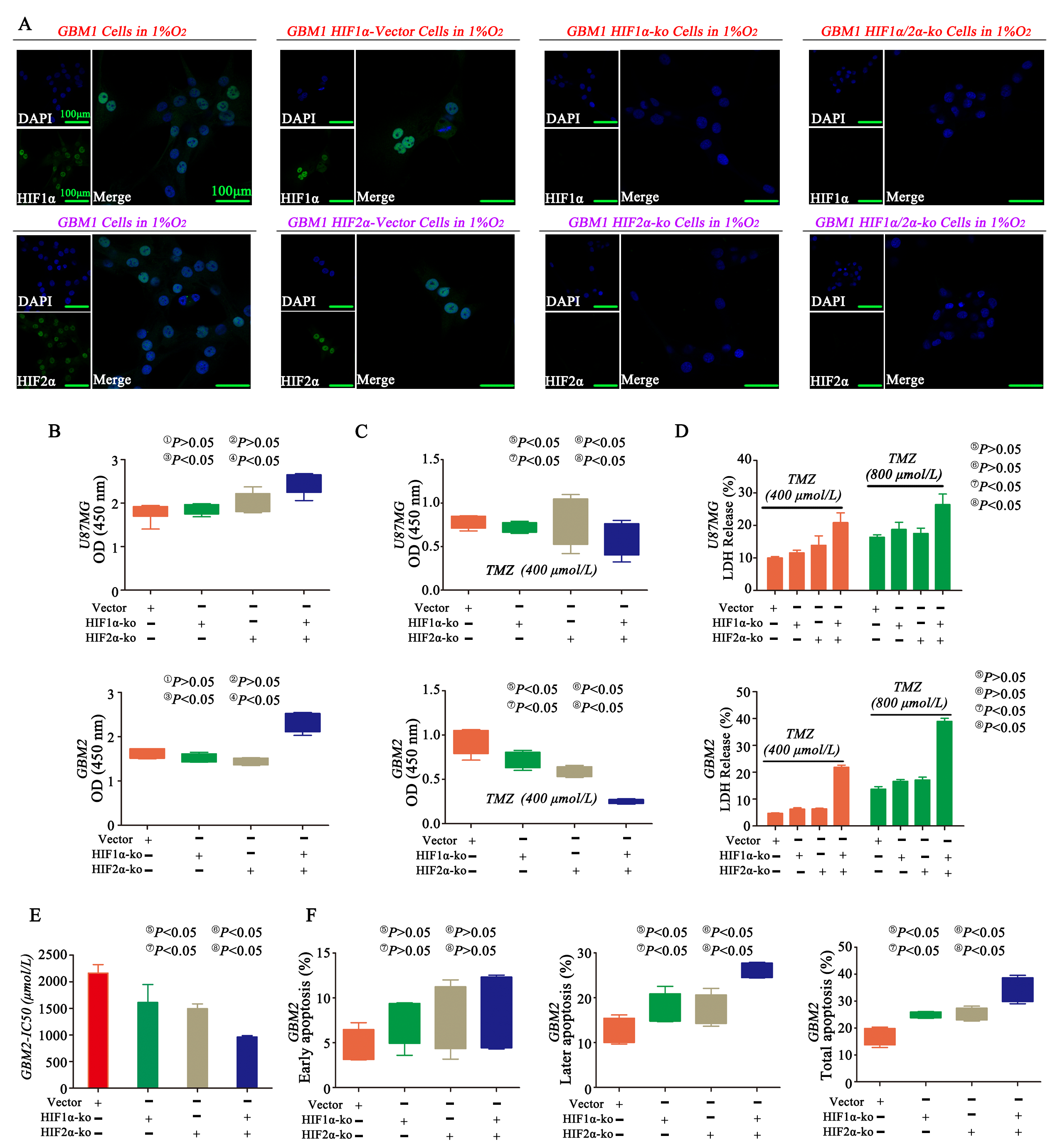

Supplement: Supplementary file 9 — Supplementary Figure 3 [file 41419_2020_3150_MOESM9_ESM.tif]

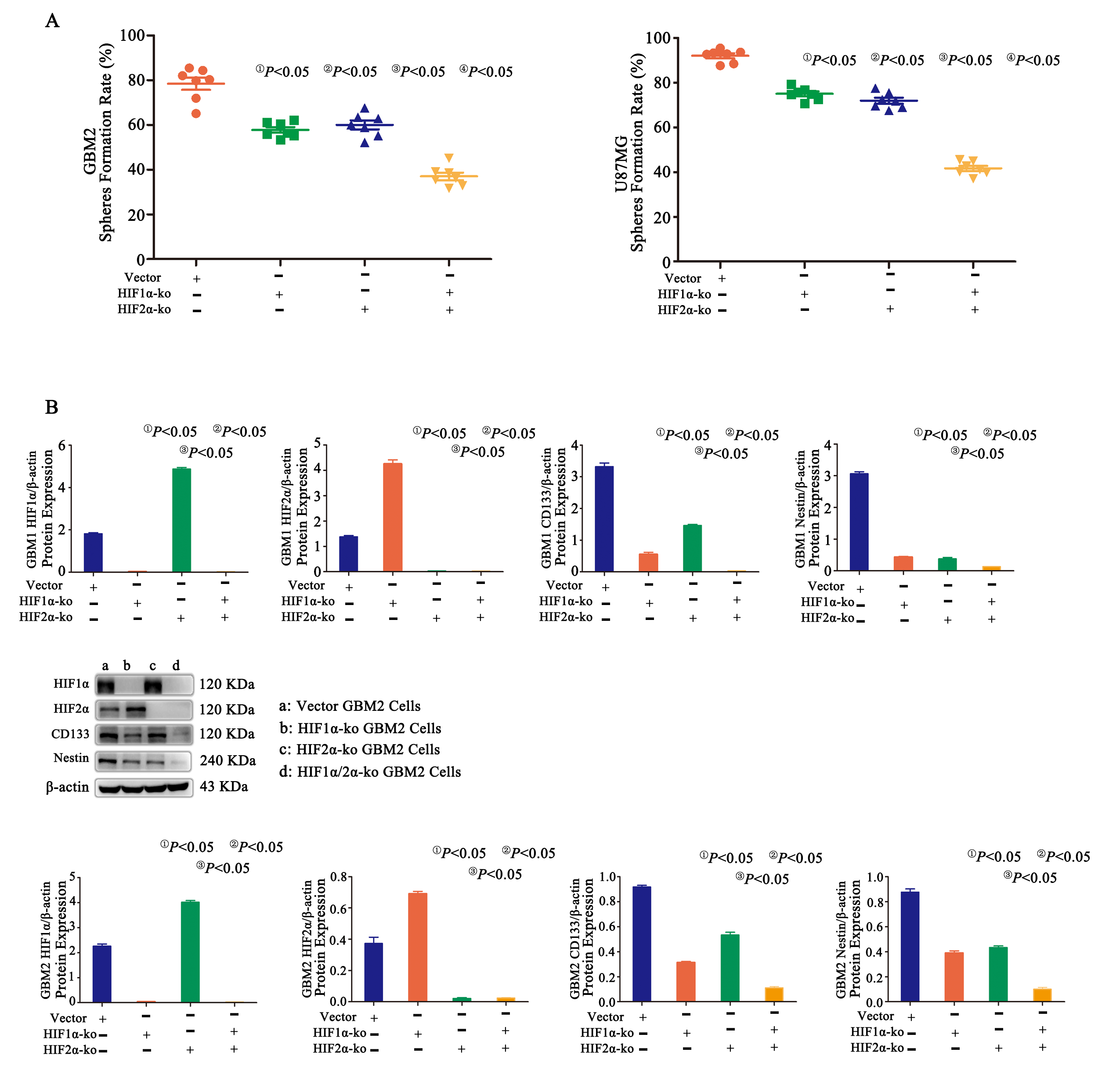

Supplement: Supplementary file 10 — Supplementary Figure 4 [file 41419_2020_3150_MOESM10_ESM.tif]

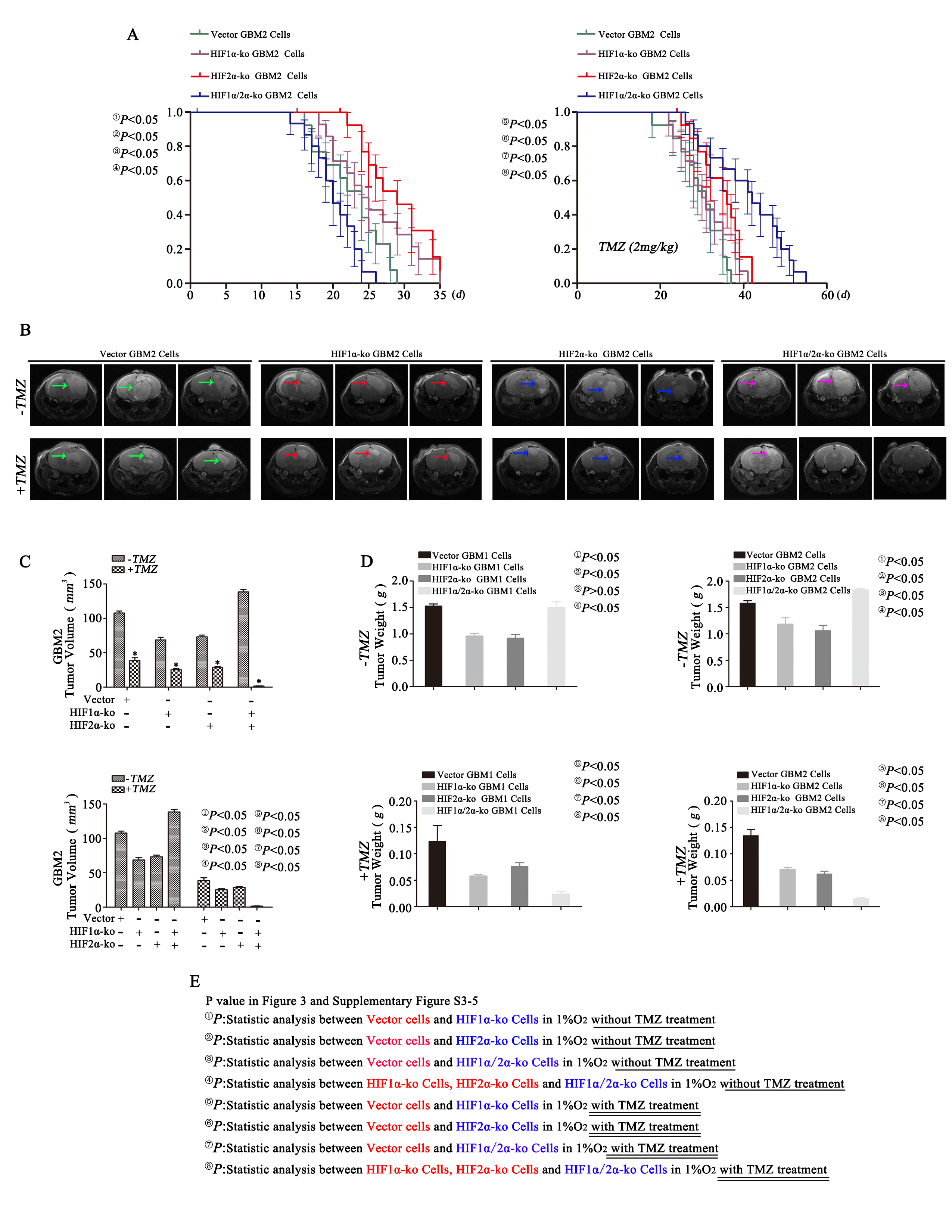

Supplement: Supplementary file 11 — Supplementary Figure 5 [file 41419_2020_3150_MOESM11_ESM.tif]

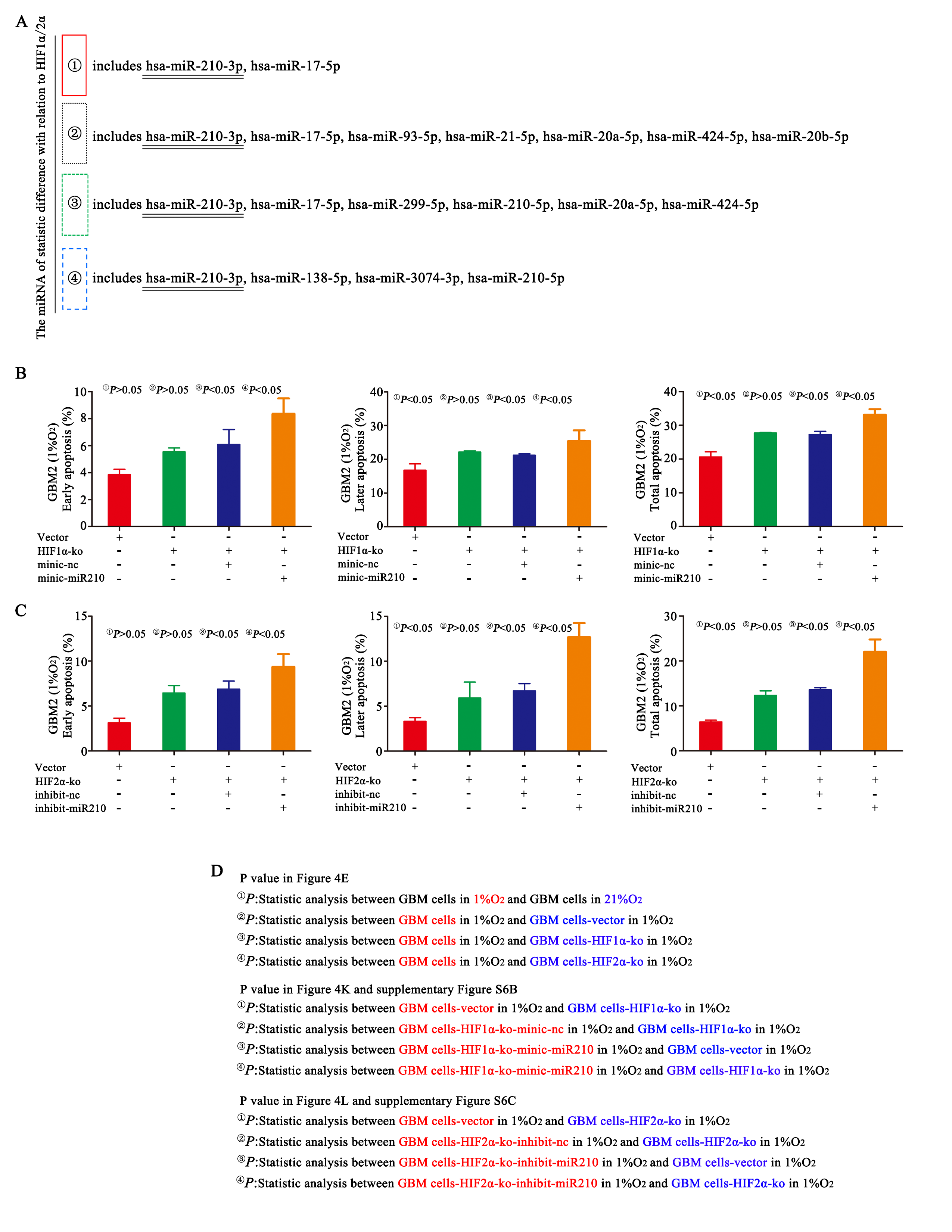

Supplement: Supplementary file 12 — Supplementary Figure 6 [file 41419_2020_3150_MOESM12_ESM.tif]

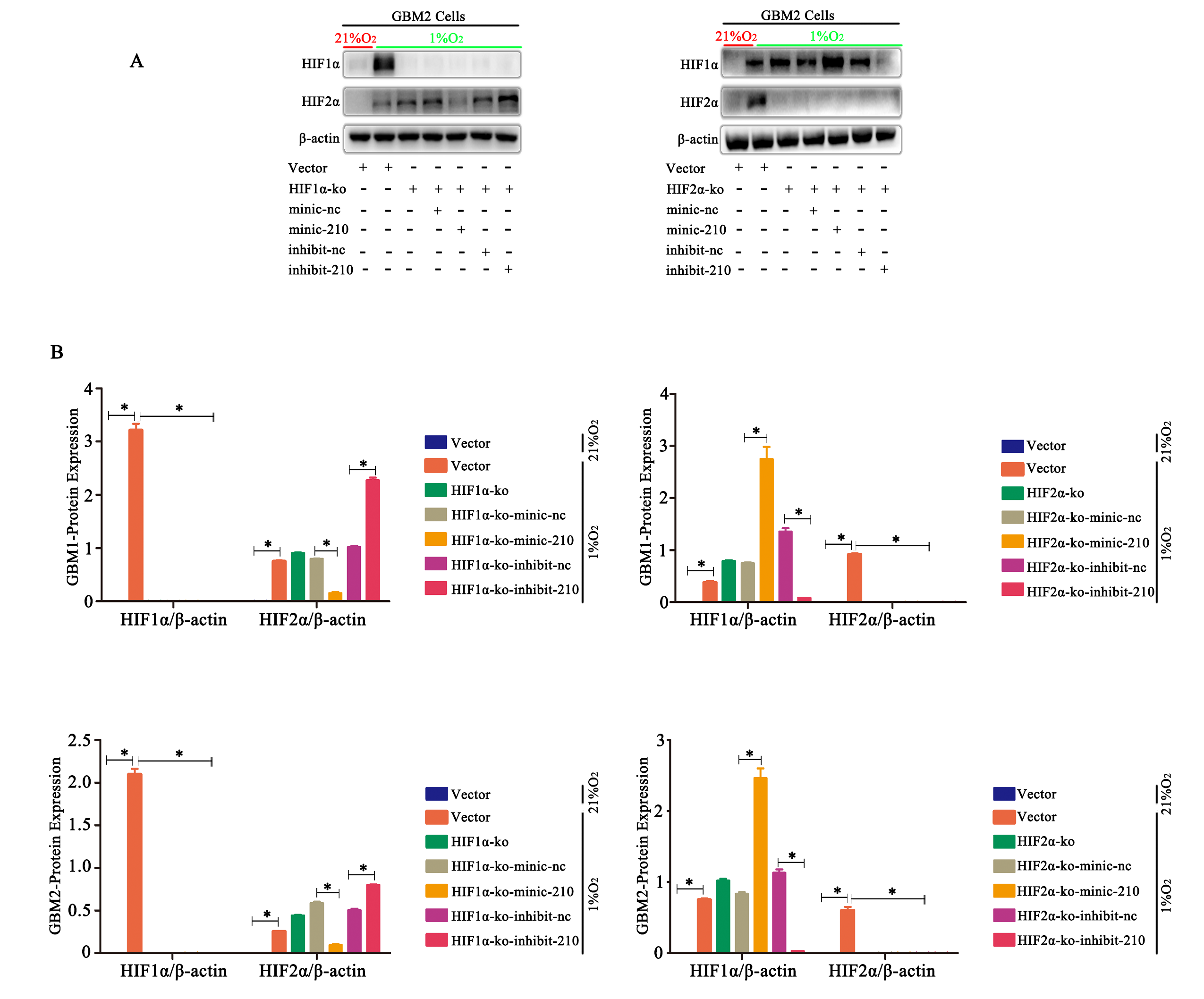

Supplement: Supplementary file 13 — Supplementary Figure 7 [file 41419_2020_3150_MOESM13_ESM.tif]

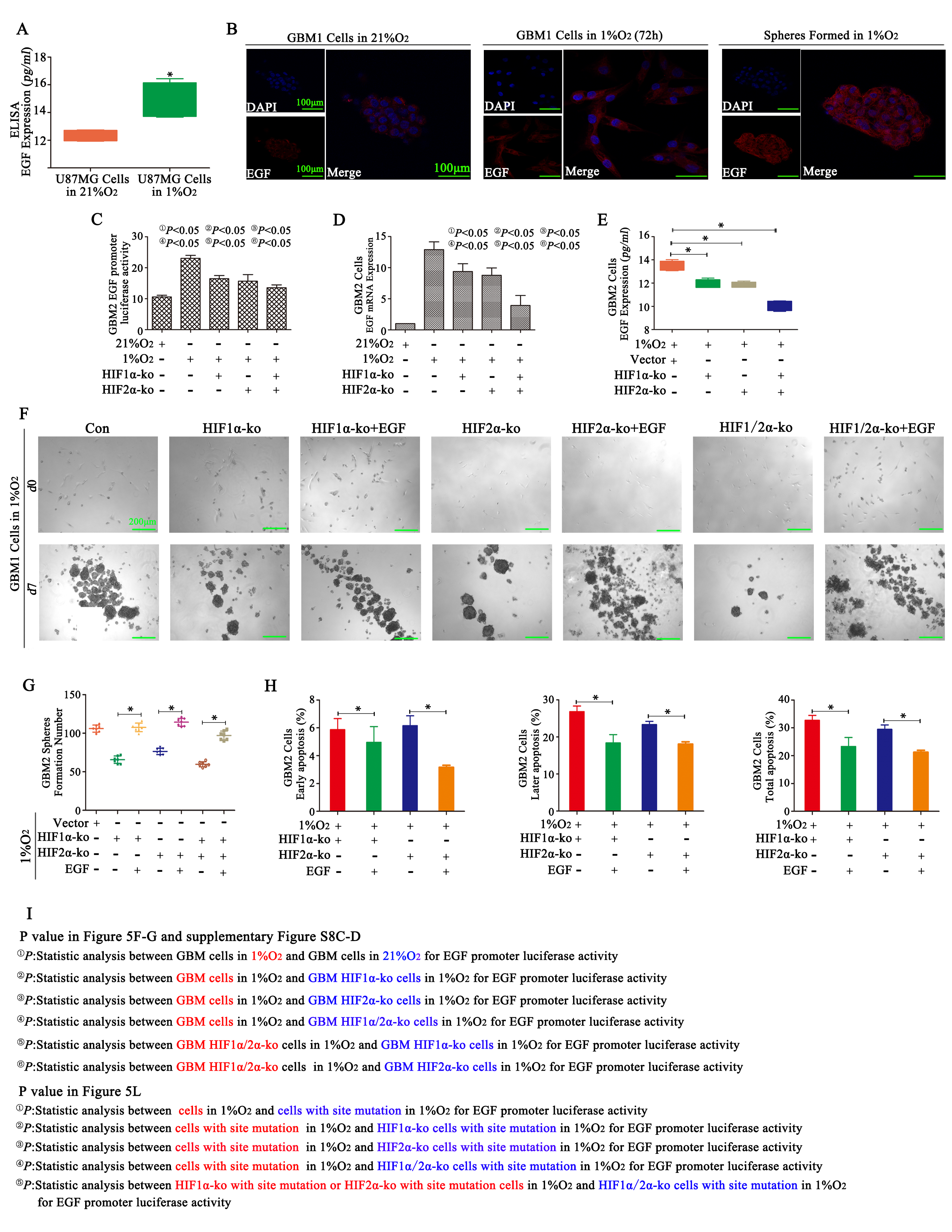

Supplement: Supplementary file 14 — Supplementary Figure 8 [file 41419_2020_3150_MOESM14_ESM.tif]
