# Supplementary material for: The HIF1α/HIF2α-miR210-3p network regulates glioblastoma cell proliferation, dedifferentiation and chemoresistance through EGF under hypoxic conditions
Source: Cell Death Dis. 2020 Nov 18;11(11):992. doi: 10.1038/s41419-020-03150-0 (PMC7674439; doi:10.1038/s41419-020-03150-0)
Supplement: Supplementary file 15 — Supplementary_Figure_Legends [file 41419_2020_3150_MOESM15_ESM.docx]

**Supplementary Figure 1. S**[**tatistical**](javascript:;) [**analysis**](javascript:;) **of HIF1α and HIF2α expression. A** Immunofluorescence staining revealed higher HIF1α expression in GBM1 cells cultured under hypoxic conditions for 48 h than in cells cultured under hypoxic conditions for 6 h, but the difference in HIF2α expression was not significant. **B** Western blot showing a time-dependent increase in HIF1α levels in cells cultured under hypoxic conditions, but the difference in HIF2α expression was not significant. **C** HIF1α was expressed at higher levels in tumour tissues than in normal tissues; however, a significant difference in HIF2α expression was not observed between tumour and normal tissues. **D** From WHO grade II to IV, HIF1α expression increased significantly, but HIF2α levels remained similar. ^*^*P*<0.05 and ^#^*P*>0.05 were determined using Student’s t test or one-way analysis of variance.

**Supplementary Figure 2. Hypoxia promoted G_1_ phase arrest and inhibited apoptosis. A** GBM2 cells were cultured in the presence of 1% O2 or 21% O2, and the results revealed a higher percentage of cells in G1 phase after exposure to 1% O2 compared with cells cultured in the presence of 21% O2. **B** The addition of TMZ (0, 100, 200, 400 and 800 μM) to the medium of GBM2 cells resulted in lower levels of LDH release under hypoxic conditions than under control conditions. **C** GBM2 cells were exposed to TMZ (400 μM) for 72 h in the presence of different oxygen concentrations and higher percentages of late and total apoptotic cells were observed after culture under normoxic conditions compared with hypoxic cells, but no difference in the percentage of early apoptotic cells was observed. **D** Higher IC50 values were observed in GBM2 cells cultured under hypoxic conditions than in the cells cultured under normoxic conditions. ^*^*P*<0.05 and ^#^*P*>0.05 were determined using Student’s t test.

**Supplementary Figure 3. HIF1α and HIF2α regulated cell proliferation and apoptosis. A** Immunofluorescence staining confirmed successful HIF1α and HIF2α knockout in GBM1 cells. **B** The CCK-8 assay did not reveal significant differences in the proliferation of GBM2 and U87MG cells with single HIF1α or HIF2α knockout in the absence of the TMZ treatment; however, the proliferation rate increased significantly after simultaneous HIF1α and HIF2α knockout. **C** GBM2 and U87MG cells with simultaneous HIF1α and HIF2α knockout showed the lowest proliferation rate after TMZ treatment (400 μmol/L) for 72 h compared with the control. **D** Higher levels of LDH release were observed in GBM2 and U87MG cells with simultaneous HIF1α and HIF2α knockout after TMZ treatment compared with the other groups. **E** Lower IC50 values were observed after HIF1α or HIF2α knockout; and the lowest IC50 value was detected after simultaneous HIF1α and HIF2α knockout. **F** Higher percentages of late and total apoptotic cells were observed after HIF1α or HIF2α knockout; and the highest percentages of late and total apoptotic cells were observed after simultaneous HIF1α and HIF2α knockout. *P* values were determined using one-way analysis of variance.

**Supplementary Figure 4. HIF1α and HIF2α regulated cell stemness. A** The sphere formation rate by a single GBM2 or U87MG cell cultured in the presence of 1% O_2_ decreased after HIF1α or HIF2α knockout, and the lowest rate was observed after simultaneous HIF1α and HIF2α knockout. **B** Western blots showing increased HIF1α levels after HIF2α knockout, increased HIF2α levels after HIF1α knockout, and significantly decreased levels of CD133 and Nestin after HIF1α and HIF2α knockout in GBM1 and GBM2 cells. *P* values were determined using one-way analysis of variance.

**Supplementary Figure 5. HIF1α and HIF2α regulated the tumorigenesis of GBM cells. A** The cells listed above were injected into the mouse brain, and an analysis of the survival time showed that HIF1α or HIF2α knockout alone in the absence of the TMZ treatment correlated with a longer survival time than the control, but simultaneous HIF1α and HIF2α knockout correlated with a shorter survival time than the control. However, after TMZ exposure, the group with both HIF1α and HIF2α knockout showed the longest survival time compared with the survival time of the other three groups. **B-D** GBM2 cells with simultaneous HIF1α and HIF2α knockout produced tumours with a larger volume and weight than control cells without HIF1α and HIF2α knockout, and the tumours of both groups described above were larger than in groups with single HIF1α or HIF2α knockout. The intraperitoneal injection of TMZ (2 mg/kg) into the aforementioned groups reduced the tumour volume and weight after HIF1α or HIF2α knockout, and the smallest tumour volume and weight were observed in the group with dual HIF1α and HIF2α knockout. **E** *P* values for the data presented in Figure 3 and Supplementary Figures S3-5. *P* values were determined using one-way analysis of variance, and the survival time was analysed using the log-rank test..

**Supplementary Figure 6. miR-210-3p regulated HIF1α and HIF2α expression in hypoxic cells. A** Significantly differentially expressed miRNAs in relation to HIF1α and HIF2α expression in each group. **B-C** The overexpression of miR-210-3p in HIF1α-KO cells or silencing of miR-210-3p expression in HIF2α-KO cells led to a higher percentage of apoptotic cells. **D** The specific *P* values for the data presented in Figure 4E, 4K, 4L and Supplementary Figure S6B-C are shown. *P* values were determined using one-way analysis of variance.

**Supplementary Figure 7.** HIF1α-KO GBM2 cells were cultured in the presence of 1% O_2_, and miR-210-3p was overexpressed or inhibited. HIF2α levels decreased with miR-210-3p overexpression and increased when miR-210-3p expression was inhibited. Then, HIF2α-KO GBM2 cells were cultured in the presence of 1% O_2_, and miR-210-3p was overexpressed or inhibited. HIF1α expression increased with miR-210-3p overexpression and decreased with the inhibition of miR-210-3p expression. ^*^*P*<0.05 was determined using Student’s t test.

**Supplementary Figure 8. High expression of EGF in hypoxic cells increased stemness. A** ELISA showing high EGF levels in U87MG cells cultured in the presence of 1% O_2_ for 72 h. **B** Immunofluorescence staining showing high EGF expression in the spheres and GBM1 cells exposed to 1% O_2_ for 72 h; however, low levels of EGF expression were observed in cells exposed to 21% O_2_. **C-E** Lower EGF promoter activity and expression of the EGF mRNA and protein were observed in the cells with single HIF1α or HIF2α knockout than in the control cells, but all the cells listed above displayed higher EGF promoter activity than cells with dual HIF1α and HIF2α knockout. **F** Images of HIF1α- or HIF2α-KO cells cultured in the presence or absence of EGF. **G** The number of spheres increased after the addition of EGF to the culture medium of HIF1α- or HIF2α-KO GBM2 cells cultured in the presence of 1% O_2_. **H** A decreased percentage of apoptotic cells was observed after the addition of EGF to the culture medium of all groups. **I** The specific *P* values for the data presented in Figure 5F-G, L and Supplementary Figure S8C-D are shown. ^*^*P*<0.05 was determined using Student’s t test and one-way analysis of variance.

**Supplementary Table S1** The differentially expressed miRNAs identified among control cells, HIF1α-KO cells, HIF2α-KO cells, and HIF1α/HIF1α-KO cells cultured under hypoxic conditions.

**Supplementary Table S2** The sequences of primers used for RT-qPCR detection.

**Supplementary Table S3** The sequences of the miR-210-3p mimic and inhibitor.

**Supplementary Table S4** The sequences of sgRNAs used to knockout HIF1α and HIF2α.

**Supplementary Table S5** Primary antibodies used for western blotting and Elisa.

**Supplementary Table S6** The antibodies used for immunofluorescence staining and IHC.
