# Supplementary material for: The HIF1α/HIF2α-miR210-3p network regulates glioblastoma cell proliferation, dedifferentiation and chemoresistance through EGF under hypoxic conditions
Source: Cell Death Dis. 2020 Nov 18;11(11):992. doi: 10.1038/s41419-020-03150-0 (PMC7674439; doi:10.1038/s41419-020-03150-0)
Supplement: Supplementary file 16 — Supplementary_materials_and_methods [file 41419_2020_3150_MOESM16_ESM.docx]

**Materials and methods**

**Public data collection**

Data from TCGA, GTEx and CCLE databases were obtained at http://cancergenome.nih.gov/, <https://www.gtexportal.org/> and <https://portals.broadinstitute.org/ccle>, respectively. These data were used to analyse the correlations between the expression of HIF1α, HIF2α and other proteins with the dplyr, tibble and ggpolt2 packages in R. GEPIA (<http://gepia.cancer-pku.cn/detail.php>) was used to analyse the differences in protein expression between tumour and normal tissues from TCGA and GTEx databases. OS and DFS analyses of patients included in TCGA database were performed using GEPIA.

**Patients and specimens**

Fifteen glioma tissues (WHO II, four tissues; WHO III, four tissues; WHO IV, seven tissues) were obtained during surgery, and the grade was verified pathologically after surgery. Different grades of glioma tissues were used to detect mRNA and protein expression using RT-qPCR and western blotting, respectively. The tumour tissues obtained from patients during surgery were anonymized.

**Cell isolation and cell culture**

U87MG cells were obtained from ATCC, and primary glioblastoma (GBM) cells were isolated from the tissues collected after surgery in this study. The primary GBM tissues were initially minced, and the tissues were digested with 0.25% trypsin (HyClone, USA) and 10 U mL^−1^ DNase I (Sigma, USA) at 37°C for 45-60 min. The red blood cells were lysed with ACK lysis buffer (Beyotime Biotechnology, China). The suspension was washed and filtered through a 100-μm cell strainer. The cells were cultured in DMEM/F12 (HyClone, USA) supplemented with 10% foetal bovine serum (FBS, Gibco, USA) to maintain growth in 21% O_2_ and 5% CO_2_ at 37°C. U87MG cells were authenticated by STR profiling and all the cells were verified none mycoplasma contamination.

**Clonogenicity and asymmetric division assays**

GBM and U87MG cells were digested with 0.25% trypsin, suspended in DMEM/F12+10% FBS and centrifuged. The cells were resuspended again in DMEM/F12+10% FBS and diluted to a density of 1500 cells/1 mL of DMEM/F12+10% FBS. One microliter of the mixed medium was placed in each well of 96-well plates, which contained 170 μL of serum-free DMEM/F12. Six 96-well plates were established for each cell line, and these plates were randomly divided into two groups: one was incubated at 37°C with 1% O_2_ and the other was incubated at 37°C with 21% O_2_. The cell states were observed, imaged and recorded at 3, 7, 14 and 21 days. The newly formed spheres were centrifuged; one group was cultured with stem cell medium (DMEM/F12+EGF+FGF2+B27) and the other group was incubated with differentiation culture medium (DMEM/F12+10% FBS). Both groups were cultured at 37°C in the presence of 21% O_2_, and the cell state was observed, imaged and recorded on days 1, 3 and 5.

**Immunofluorescence staining**

GBM and U87MG cells were cultured in the presence of 1% O_2_ for 48 h or 72 h, and protein expression in the spheres that formed in the presence of 1% O_2_ was detected using immunofluorescence staining. These cells were initially fixed with 4% paraformaldehyde at 4°C for 30 min, washed twice with PBS containing 0.5% Triton X-100 (Sigma, USA), and then blocked with 10% normal serum. These cells were further incubated with primary antibodies against CD133, CD15, Nestin, ABCG2, HIF1α, HIF2α and EGF (the related information about the primary antibodies is presented in Supplementary Table S6) for 24 h at 4°C. The cells were washed with PBS at least three times for 5 min each and incubated with fluorophore-labelled secondary antibodies (CST, USA) for 2 h at 37°C. The images were obtained with a laser scanning confocal microscope (LSM780, ZEISS, Germany).

**Western blot detection**

Primary GBM tissues, GBM and U87MG cells were cultured under normoxic or hypoxic conditions for protein detection using western blot analysis. Total proteins were prepared from primary GBM tissues, GBM and U87MG cells using prechilled RIPA buffer (Beyotime Biotechnology, China). The cells were separated on SDS-PAGE gels and transferred to nitrocellulose membranes, which were blocked with 5% non-fat milk and incubated with antibodies against CD133, Nestin, HIF1α and HIF2α (the related information for primary antibodies is presented in Supplementary Table S5) at 4°C overnight. HRP-labelled secondary antibodies (Beyotime Biotechnology, China) were added to the blocking buffer and incubated with the membrane at 37°C for 1 h. Enhanced chemiluminescence was used for visualization.

**Real-time quantitative polymerase chain reaction**

Primary GBM tissues and GBM cells cultured in the presence of 1% O_2_ were used to detect RNA expression with RT-qPCR. Total RNA was prepared from primary GBM and U87MG cells using TRIzol (Invitrogen, USA) according to the manufacturer’s instructions. Melting occurred at 94°C for 5 min, denaturation occurred at 94°C for 30 s, annealing occurred at 57°C for 30 s, and extension occurred at 72°C for 30 s for a total of 40 cycles. The primer sequences are presented in Supplementary Table S2.

**Flow cytometry (FCM) analysis**

FCM was used to detect the cell cycle of GBM and U87MG cells cultured in the presence of 21% O_2_ or 1% O_2_ without TMZ treatment. In addition, the cells were exposed to TMZ (400 μM) and cultured in the presence of 1% O_2_ for an additional 72 h to detect cell apoptosis. Detailed descriptions of the procedures for the cell cycle and apoptosis analyses are provided below. For cell cycle detection, the cells were digested with 0.25% trypsin and prepared as a single-cell suspension in PBS at 4°C. Cells were suspended at a density of 5 × 10^5^ cells/mL and then fixed with 75% ethanol at 4°C for 24 h. The centrifuged cells were washed with PBS, suspended in 1 mL of propidium iodide (PI) staining solution and incubated at 37°C for 30 min. For cell apoptosis detection, the cells were digested with 0.25% trypsin and prepared as a single-cell suspension in PBS at 4°C. The cells were suspended at a density of 1×10^6^ cells/mL, and 100 μL of the suspension were added to an Eppendorf tube. The cells were centrifuged, and 195 μL of 0.05% trinatriumcitrate-dihydrate and 5 μL of Annexin V-FITC were added to the cell suspension and incubated for 15 min in the dark at room temperature (RT). Then, 190 μL of 0.05% trinatriumcitrate-dihydrate and 10 μL of PI were added to the centrifuged cell suspension and incubated for 10 min in the dark at RT. Both cell cycle and apoptosis samples were analysed using FACS (BD Accuri C6, Germany).

**LDH release assay**

The LDH release assay kit was purchased from Beyotime Biotechnology, and LDH release was detected according to the manufacturer’s protocol. Briefly, suspended cells were pipetted and washed with PBS, followed by the addition of DMEM/F12+1% FBS, and the cell suspension was prepared again. Cells were seeded in 96-well plates at a density of 5×10^4^ cells in 100 µL of media and cultured with TMZ in the presence of 21% O_2_ or 1% O_2_ for 72 h. The cell suspension was centrifuged at 400 *g* for 5 min. One hundred twenty microliters of the supernatant and 60 μL of the LDH test fluid were placed in 96-well plates and incubated for 30 min at 25°C in the dark. LDH release was then detected using an ELISA plate reader (Varioskan Flash, Thermo Scientific, USA) at 490 nm.

**IC50 determination**

Primary GBM and U87MG cells were cultured in 96-well plates (5,000 cells/well), cultivated in 21% O_2_ or 1% O_2_ for 72 h, and exposed to TMZ (0, 250, 500, 1,000 and 2,000 μM) for another 48 h. Next, 10 µL of CCK-8 solution with 90 µL of DMEM/F12+10% FBS were added to the culture, followed by the addition of 100 µL of the suspension into each well and culture of the suspensions in the presence of 21% O_2_ or 1% O_2_ for an additional 2 h. The OD values were then measured using an ELISA reader at 450 nm (Varioskan Flash, Thermo Scientific, USA). SPSS 19.0 software was used to calculate the IC50 values.

**CCK-8 assay**

The CCK-8 assay was performed to examine cell proliferation in the absence or presence of TMZ (400 μM). Primary GBM cells were plated in 96-well plates (2,000 cells/well) with DMEM/F12+10% FBS and cultivated in the presence of 21% O_2_ or 1% O_2_, and cell growth was detected on days 1, 2, 3, 4, 5, 6 and 7. For each cell line, four 96-well plates were established. Two plates were cultured in the presence of 21% O_2,_ and the others were exposed to 1% O_2_. Ten microliters of CCK-8 and 90 µL of DMEM/F12+10% FBS were mixed, and the mixture was added to each well. The samples were cultured for an additional 2 h in the presence of 21% O_2_ or 1% O_2_. The absorbance was measured with an ELISA reader (Varioskan Flash, Thermo Scientific, USA) at 450 nm.

**EGF ELISA**

Primary GBM and U87MG cells were seeded in 6-well plates at a density of 2×10^5^ cells per well in DMEM/F12+10% FBS and incubated in the presence of 21% O_2_ or 1% O_2_ for 72 h. The conditioned media were removed, and the cells were stored at -80°C and then counted. The EGF concentration in this media was detected with a commercially available ELISA kit (R&D Systems).

**Prediction of the HIF1α and HIF2α binding sites in the EGF sequence**

The hypothetical HIF1α and HIF2α binding sites in the EGF promoter sequence were predicted using <http://jaspar.genereg.net/>, which revealed that the EGF promoter might be complementary to the seed sequences of HIF1α and HIF2α.

**Detection of luciferase activity of the EGF promoter**

The promoter of the human EGF gene was cloned into a pGL3-basic luciferase reporter vector, and mutations were introduced at the HIF1α and HIF2α binding sites (Sangon, Shanghai, China). The normal or mutated EGF promoter reporter plasmids were cotransfected with a PRL vector (Promega) into HEK293T, HEK293T-HIF1α-KO, HEK293T-HIF2α-KO and HEK293T-HIF1α/2α-KO cells using Lipofectamine 2000 reagent. Twenty-four hours after transfection, the lysed cells were assayed using a Dual-Luciferase Assay Kit (Promega). The promoter activity was measured by comparing the luciferase levels.

**HIF knockout assays**

The plasmid constructs for human HIF1α and HIF2α sgRNAs were designed with an online CRISPR design program (http://crispr.mit.edu), annealed and cloned into the lentiCRISPRv2 vector (Addgene, #52961, USA), and the sgRNA oligonucleotide sequences are listed in Supplementary Table S4. The lentivirus was then transfected into 293T cells with the transducing vector, followed by the packaging vectors psPAX2 (Addgene #12260, USA) and pMD2.G (Addgene #12259, USA). Forty-eight hours after transfection, the supernatant containing the virus particles was collected, filtered and transduced into U87MG and GBM cells. Immunofluorescence staining or western blotting was performed to confirm the knockout of HIFs. The proliferation, cell cycle, apoptosis, LDH release, IC50 values and EGF expression in cells cultured with or without TMZ in the presence of 1% O_2_ for 24 h were detected to determine the effects of low HIF1α or HIF2α expression on glioma cells. Detailed descriptions of these methods are provided above.

**miRNA-Seq analysis**

The control cells without HIF1α and HIF2α interference, HIF1α-KO cells, HIF2α-KO cells and dual HIF1α/HIF2α KO cells were cultured under hypoxic conditions for 24 h and then the cells were collected. The cells were subjected to an miRNA-Seq analysis (GCBI, Shanghai, China). The raw data were normalized using the quantile algorithm from the limma package in R, and the raw miRNA-Seq data were uploaded in the NCBI Gene Expression Omnibus (GEO) database (www.ncbi.nlm.nih.gov/geo) under accession number GSE142719. Heat maps showing differentially regulated miRNAs were generated using Cluster 3.0 and Gene Tree View software.

**miR-210-3p detection**

According to the miRNA-Seq data, the miRNAs that targeted HIF1α and HIF2α and were commonly differentially expressed between the groups were analysed. The GBM tissues and the cells were cultured in the presence of 1% O_2_ to detect miR-210-3p expression using RT-qPCR. A pan-cancer analysis of survival times was performed using oncolnc (<http://www.oncolnc.org/>) based on TCGA survival data for miRNAs. The mimic and interference fragments were purchased from Sangon Biotechnology, Shanghai, China, and miR-210-3p was overexpressed or silenced (the sequences of the miR-210-3p mimic and inhibitor are shown in Supplementary Table S5) in HIF1α- or HIF2α-KO cells according to the manufacturer’s instructions. The cells described above were cultured in the presence of 1% O_2_, and protein expression was detected using western blotting and apoptosis was detected using FCM.

**In vivo experiment**

BALB/c-nu mice (male, 4~6 weeks) were used in this study. Primary GBM cells (8×10^4^) were seeded in the brains of 5 mice, and the mice were fed under conditions of 10% O_2_ for 14 days. The tumour tissues and normal tissues were collected to analyse the expression of HIF1α and HIF2α using RT-qPCR, western blotting and immunohistochemistry (IHC). The tissues from mice housed in a hypoxic environment were also subjected to Hypoxyprobe detection. The detailed procedures used for RT-qPCR and western blot are described above, and the methods for IHC and Hypoxyprobe detection are described below.

Control cells without HIF1α and HIF2α interference, HIF1α-KO cells, HIF2α-KO cells and dual HIF1α/HIF2α-KO cells (8×104) were injected into the brains of the mice. The mice were fed in a room containing 10% O2 and were randomly grouped into 8 groups with 25 mice per group (Con, Con+TMZ, HIF1α-KO, HIF1α-KO+TMZ, HIF2α-KO, HIF2α-KO+TMZ, HIF1α/HIF2α-KO, and HIF1α/HIF2α-KO+TMZ). Simple size for each group was estimated by (μ_α_+μ_β_)^2^×*p*_0_×(1- *p*_0_)/(*p*-*p*_0_)^2^. TMZ (2 mg/kg) was injected into the enterocoelia daily from days 3 to 17 (lasting for two weeks). MRI was used to detect the tumour volume in 5 randomly selected mice on day 21. Tumour tissues were collected from another 5 mice and protein expression was analysed using IHC, RT-qPCR and western blotting, as described above. The remaining mice were used to observe and record the survival times, and the dead mice were excluded after implantation just three day since it was due to anaesthesia or other complications. The ethics committee of Southwest Hospital at Army Medical University approved all animal procedures.

**Immunohistochemistry (IHC) detection**

We compared HIF1α and HIF2α expression between tumour tissues and normal tissues using IHC. Briefly, the tissue sections were initially fixed with formalin and embedded in paraffin, followed by dewaxing in xylene, rinses with a graded series of ethanol solutions and rehydration in double-distilled water. For antigen retrieval, the slides were pretreated with sodium citrate buffer for 15 min at 95°C. The slides were washed with PBS for 3 min and immunostained with primary antibodies against HIF1α and HIF2α at 4°C overnight. The slides were washed with PBS buffer again, and the tumour and normal tissue sections were covered with HRP-conjugated anti-mouse/rabbit antibodies for 2 h. The tissue samples were covered with the DAB chromogen solution, incubated for ~1 min to allow the staining reactions to occur, and the images were captured.

**Detection with the Hypoxyprobe^TM^-1 kit**

The antihuman Hypoxyprobe^TM^-1 kit was purchased from Hypoxyprobe (Burlington, MA, USA). Then, 60 mg/kg Hypoxyprobe^TM^-1 was intraperitoneally [injected](javascript:;), and the mice were fed for an additional 1 h. Mouse brain tumour tissues were fixed with 4% paraformaldehyde, embedded in paraffin, sliced and stained using IHC. Then, 100 μmol/mL Hypoxyprobe^TM^-1 was added to the cultured GBM cells for 1 h, and the cells were fixed with 4% paraformaldehyde for 30 min and subjected to immunofluorescence staining.

**Statistical analysis**

SPSS 19.0 software was used for statistical analyses. Data are presented as the means±standard deviations (SD). Student’s t test was used to assess the significance of differences between the two groups, and one-way analysis of variance (one-way ANOVA) was used to compare data from at least three groups. The log-rank test was used to analyse the OS or DFS. Pearson’s correlation coefficient was calculated to analyse the correlations between gene expression levels. *P*<0.05 was considered a statistically significant difference.
